# Optimizing Nanofiltration Membrane Layer-by-Layer Modification: Chemometric and Morphological Insights

**DOI:** 10.1021/acsapm.5c00815

**Published:** 2025-06-26

**Authors:** Tanaz Moghadamfar, Rodrigo Rocha de Oliveira, José Luis Cortina, Luis J. del Valle, Anna de Juan, Mònica Reig

**Affiliations:** † Chemical Engineering Department, Escola d’Enginyeria de Barcelona Est (EEBE), Universitat Politècnica de Catalunya (UPC)-Barcelona TECH, Campus Diagonal-Besòs, 08930 Barcelona, Spain; ‡ Barcelona Research Center for Multiscale Science and Engineering, Campus Diagonal-Besòs, 08930 Barcelona, Spain; § Department of Chemical Engineering and Analytical Chemistry, Chemometrics Group, 16724 Universitat de Barcelona, Martí I Franquès 1, 08028 Barcelona, Spain; ∥ CETaqua, Carretera d’Esplugues 75, 08940 Cornellà de Llobregat, Spain

**Keywords:** polyelectrolyte multilayer, composite membrane, selectivity enhancement, flat sheet membrane, Fortilife-XN

## Abstract

Nanofiltration (NF) membranes are essential in wastewater treatment, battery industries, and brine management for selectively removing multivalent ions. However, fouling reduces their lifespan and necessitates harsh cleaning. The layer-by-layer (LBL) technique addresses this by modifying surface properties, enhancing rejection of divalent cations, such as magnesium and calcium, and minimizing fouling. This study evaluated a semiaromatic-based polyamide NF membrane (Fortilife-XN) modified using a LBL technique. Surface properties such as contact angle (CA), roughness, morphology, uniformity, and thickness were analyzed before and after modification. FTIR characterization revealed the membrane’s structure, comprising a polyethylene terephthalate (PET) support, a polysulfone (PS) substrate, and a polyamide (PA) active surface. Poly­(sodium 4-styrenesulfonate) (PSS) was used as the polyanion, while poly­(diallyldimethylammonium chloride) (PDADMAC) and poly­(allylamine hydrochloride) (PAH) served as strong and weak polycations, respectively. Modifications in varying bilayers (1.5–6.5 BLs) with a positive terminal half-layer introduced peaks at 1034 and 923 cm^–1^, corresponding to sulfonate and C–N bonds. CA and roughness analysis showed that (PDADMAC/PSS)­4.5 (CA 20°, roughness 77 nm) and (PAH/PSS)­1.5 (CA 61°, roughness 79 nm) achieved superior wettability and roughness, confirmed by initial NF testing with a permeability of around 10 L/(m^2^ h bar). Ellipsometry, using Cauchy and Sellmeier models, measured multilayer thickness, estimating bilayers at ∼2 nm. Raman imaging visualized cross-sectional modifications, distinguishing raw and modified layers. Surface imaging revealed more uniform PAH deposition, while PDADMAC showed higher swelling with increased layers due to stronger affinity. These combined analytical techniques provided insights into the impact of LBL modification on membrane morphology and properties, aiding performance optimization. All membranes were preliminarily tested with a mixed salt solution (NaCl, CaSO_4_, and MgSO_4_). The average selectivity of Na/Ca for the bare membrane was 2.6 ± 0.4, increasing by 108% for (PDADMAC/PSS)­5.5 and 134% for PAH 6.5. For Na/Mg, the selectivity was 4.5 ± 0.3, rising by 49% for (PDADMAC/PSS)­5.5 and 131% for PAH 6.5.

## Graphical abstract

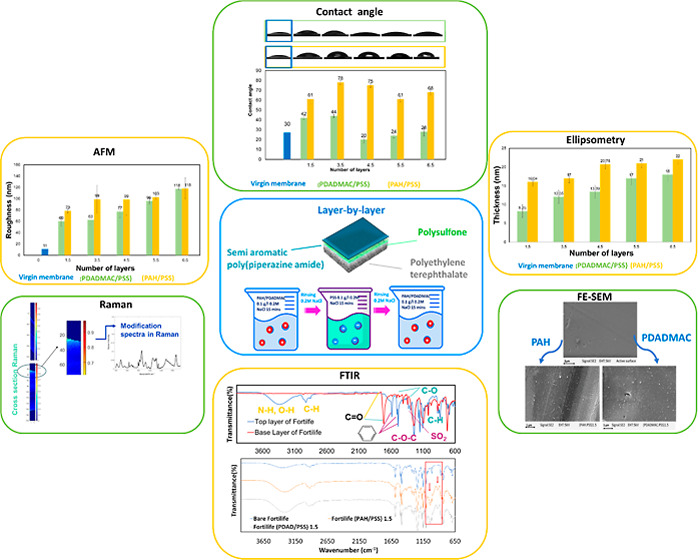

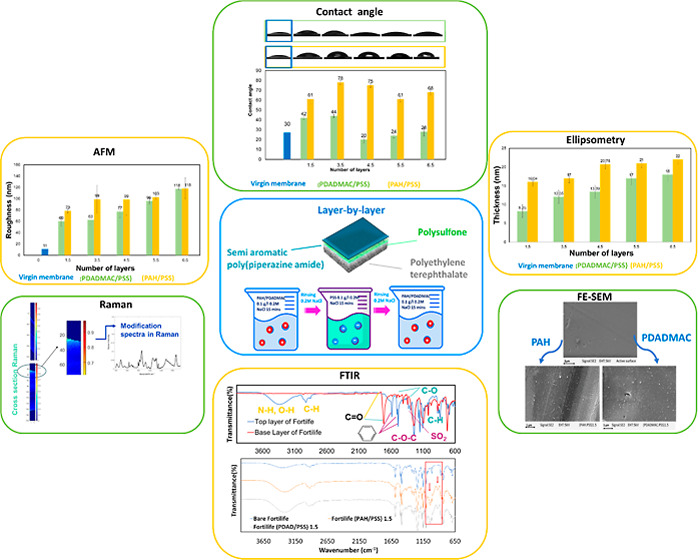

## 1 Introduction

Recent years have seen a considerable evolution in the field of industrial wastewater management, with a notable shift in focus from the removal of organic pollutants to addressing the challenges posed by inorganic solutes.^1^ The challenges posed by water scarcity in arid and semiarid regions worldwide (e.g., the USA, Australia, the Mediterranean Basin, and parts of Africa) are driving the growing necessity for the direct and/or indirect use of treated domestic and industrial wastewater. This transition is representative of a broader recognition within both urban and industrial water cycles, where pretreatment and polishing stages of membrane processes like in brine management, the battery industry, and water softening, where attention is increasingly directed toward the selective separation of inorganic components and their recovery when requested.^2^ To provide more insights, inorganic scaling reduces water flux and increases pressure in treatment systems, with calcium sulfate and magnesium phosphate as common scalants. Pretreatments, including antiscalants, help but increase costs and fouling risks. Monitoring antiscalants and using nanofiltration (NF) to remove scale-forming divalent cations can mitigate scaling effectively.^3^ Industries like textiles, paper, and drinking water rely on separating monovalent from divalent ions, which can be done by NF. For drinking water, removing calcium, magnesium, and sulfate while maintaining at least 500 mg/L TDS is essential for health, making hardness ion separation crucial.^4^

NF membranes with free volumes of 5 to 10 Å stand out as intriguing due to their unique ability to selectively reject multivalent ions while facilitating high permeability and passage of monovalent ions.^5^ This characteristic lends itself to operating under low-pressure conditions, rendering it energy-efficient and cost-effective.^6^ In NF membranes, the rejection of ions relies on dielectric exclusion, Donnan (charge) exclusion, and size (steric) exclusion. Dielectric exclusion, driven by differences in the dielectric constants, is especially crucial. Multivalent ions are rejected more effectively due to their larger size and higher charge compared with monovalent ions. It may be possible to maintain high separation by having high selectivity for the passage of monovalent ions over divalent ions.^7^ In many industrial applications today, including water purification, wastewater treatment, and desalination, the thin film composite (TFC) NF membrane has emerged as a commonly used filtration method. Typically, TFC membranes consist of three layers: a bottom porous support layer, a highly permeable middle layer, and a thin (<1 μm) species-selective top layer.^8^ Thin film composite membranes made of aromatic and semiaromatic polyamides make up the majority of NF commercial membranes. The thin film (active layer) is typically created via the interfacial polymerization of an organic trimethyl chloride solution and an aqueous *m*-phenylene-diamine solution. However, additional amine monomers can be utilized as well, including polyethylenimine, *N*-(2-aminoethyl)-piperazine, *N*-(*N*′-diamino piperazine), piperazine, and triethylenetetramine.^9^

Further advancements are vital to meet the diverse challenges in medium- and high-salinity urban and industrial wastewater treatment by fabricating positively charged NF membranes. For several reasons, positively charged NF membranes have great potential for enhancing membrane performance.^10^ Besides, positively charged NF membranes repel scalants like calcium sulfate, calcium carbonate, and magnesium phosphate, reducing fouling, extending lifespan, and improving efficiency.^11^ In addition, positively charged NF membranes reject divalent cations like calcium and magnesium more effectively, making them ideal for applications like water softening.^4^

Moreover, surface modification enhances membrane properties, improving hydrophilicity, biocompatibility, and performance in treating saline wastewater.^12^ Available techniques for NF modification are interfacial polymerization (IP), ultraviolet (UV) treatment, electron beam (EB) irradiation, plasma treatment, and layer-by-layer (LBL) modification.^3^ Out of all of the researched methods, the layer-by-layer (LBL) technique appears to be one of the most suitable and simple ways to assemble positively charged NF membranes. The LBL assembly process enables the creation of ultrathin, defect-free films with customizable compositions and adjustable properties. Polyelectrolyte multilayer (PEM) nanofiltration membranes are formed by alternating the deposition of polycations and polyanions onto a porous substrate, followed by rinsing to remove weakly linked polymer chains.^13^ The essential advantage of the LBL method lies in its precise control over the nanometer-scale thickness, determined by the number of deposition cycles. In membrane separation, the LBL approach offers precise control over film thickness, allowing the regulation of membrane selectivity and flux by adjusting the number of deposition cycles and the chemical composition of polyelectrolytes (PEs) used. Consequently, polyelectrolyte multilayer membranes (PEMMs) hold promise for creating separation membranes with high flux and remarkable selectivity.^10,14–18^ Several coating conditions can be used to customize the development and functionality of LBL membranes. The concentration, kind, and charge density of PE and salt, the number of bi-layers, and the terminating layer are a few of the tuning parameters that affect coating (explanation in Supporting Information A–G).^19^

The main objective of this study was to assess the effect of LBL modification on the active surface of a semiaromatic polyamide-based NF membrane through a comprehensive characterization analysis and to understand how they evolve in response to variations in the number of layers. The modification was explicitly aimed at enhancing membrane selectivity with respect to divalent and monovalent cations and improving overall NF membrane life time. In this study, the conventional and fundamental characterization analyses for modified membranes like FTIR spectroscopy, CA, FE-SEM, and AFM were complemented by ellipsometry and Raman image acquisition, two techniques with high novelty in this field of application. Indeed, to the best of the authors’ knowledge, no prior characterization has been conducted on Fortilife-XN as a semiaromatic polyamide membrane. Additionally, the multilayer thickness was directly measured on the raw membrane surface, while Raman imaging, used for the first time for this purpose, analyzed cross-sectional modifications and assessed their uniformity. Then the bare and modified membranes were tested with a mixed salt solution (NaCl, CaSO_4_, and MgSO_4_) to evaluate their performance in terms of permeability and selectivity when using NF.

## 2 Materials and Methods

### 2.1 Materials

The TFC NF membrane (FilmTec Fortilife-XN) was provided by the Dupont filtration company. As specified in the datasheet, the membrane is classified as a polypiperazine-based thin-film composite.^20^ It exhibits comparatively high permeability, enabling enhanced water recovery and selective passage of monovalent ions while demonstrating effective rejection of divalent ions (data provided by the supplier^21^). Sodium chloride (NaCl; 99%), glycerol (≥99%), and isopropanol (≥99.5%) were purchased from J.T. Baker. The polycations poly­(diallyl dimethylammonium chloride) (PDADMAC; Mw = 200–350 kDa, 20 wt % in water) and poly­(allylamine hydrochloride) (PAH; Mw = 50 kDa) and the polyanion poly­(styrene sulfonic acid) sodium (PSS; Mw = 70 kDa) were purchased from Merck (see Figure 1a).

**Figure 1:**
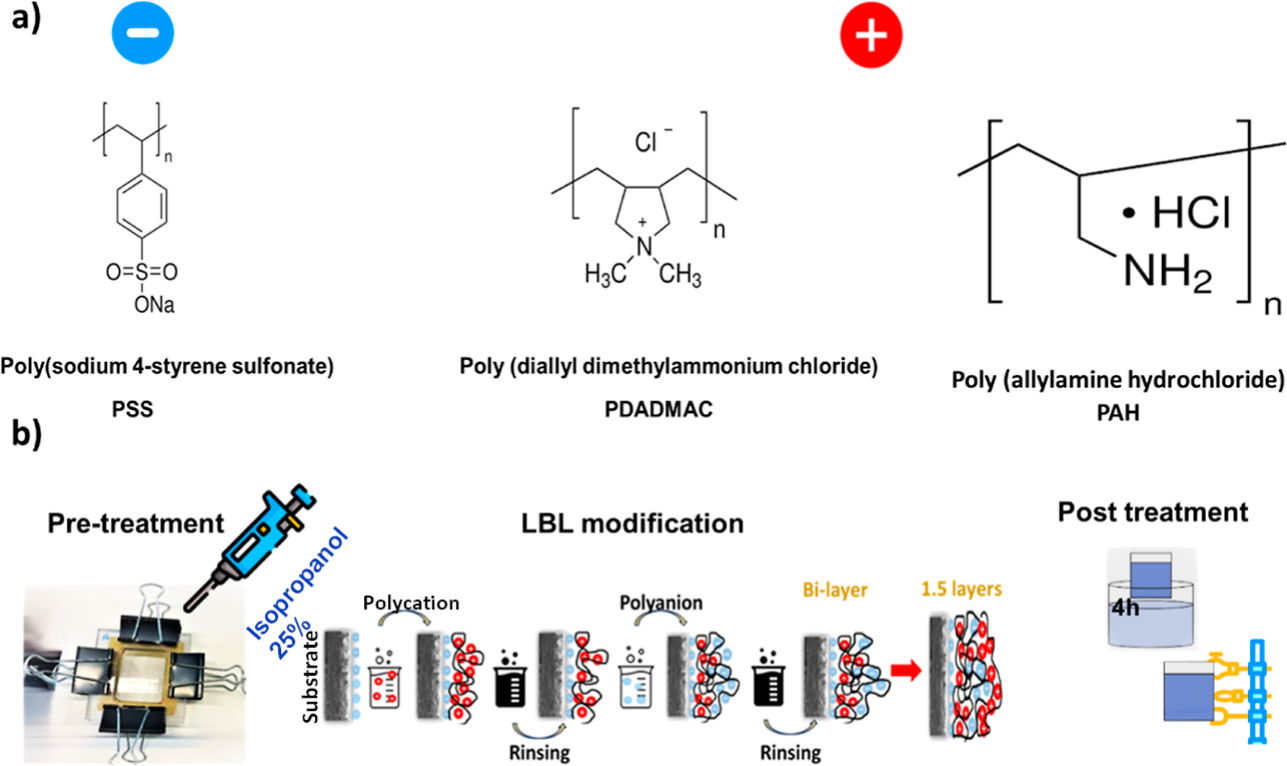
(a) The chemical composition of polyelectrolytes used: PSS (polyanion), PDADMAC (strong polycation), and PAH (weak polycation). (b) Layer-by-layer (LBL) modification procedure describing the formation of 1.5 BL.

### 2.2 Self-Assembly PEMM

The FilmTec Fortilife-XN (DuPont) membrane was used as a substrate for LBL modification because of its membrane chemistry. The methodology outlined in this article was adopted by recent studies, employing optimized parameters concerning the selection of polyelectrolytes, salt type, and concentration to produce positively charged PEMMs.^10,19^

Figure 1b outlines the process of fabricating the PEM membranes. The raw membrane was first pretreated with 25% isopropanol for 15 min and rinsed with deionized (DI) water to remove any potential contamination.^10^ The polyelectrolyte layers were allowed to form only on the negatively charged active layer using a homemade frame (24 cm^2^). The most popular strong polyanion for LBL modification, known as poly­(sodium 4-styrenesulfonate) (PSS), was the polyanion that was chosen for this work. One weak and one strong polycation were selected to evaluate and examine any potential outcome differences. Poly­(diallyl dimethylammonium chloride) (PDADMAC) and poly­(allylamine hydrochloride) (PAH) were the strong and weak polycations, respectively, that were used (see Figure 1a). Every polymer was dissolved at a concentration of 0.1 g L^–1^ in a 0.2 M sodium chloride solution.^10^ The pH value of the PSS solution was 6.1, that of the PDADMAC solution was 5.9, and that of the PAH solution was 5.8 (pH was not adjusted).

In summary, the semiaromatic polyamide membrane underwent dip-coating in a 0.1 g L^–1^ solution of polycation (PDADMAC or PAH) for 15 min. Following rinsing with a 0.2 M NaCl solution (pH 5.8), the membrane was dip-coated in a 0.1 g L^–1^ PSS solution to establish the initial bilayer.^10^ This deposition cycle was repeated 1.5–6.5 times to generate PEMMs containing 1.5–6.5 bilayers (BLs). The coatings were terminated with a polycation layer to generate a positively charged surface for this investigation. This could enhance the rejection of divalent cations, facilitate capillary action, and mitigate membrane fouling.^3,10,19,22,23^ Following the deposition procedure, the PEM membranes were submerged in a 15 wt % glycerol solution for 4 h and subsequently air-dried overnight, following recommendations from existing literature.^10,19^

### 2.3 Membrane Characterization and Chemometric Analysis

#### 2.3.1 Chemical Composition

Attenuated total reflection Fourier transform infrared spectroscopy (ATR-FTIR, Jasco 4700, Japan) was used to do an accurate comparative evaluation of membrane chemical structures. The measurement was repeated for every two samples of the same membrane in 2 different areas. The attenuated total reflection (ATR) crystal used in this investigation was made of diamond material, and the FTIR Jasco 4700 had a spectrum range of 350–4400 cm^–1^. This advanced analytical method made it possible to precisely characterize the membranes’ functional groups, providing vital information about their molecular structures and easing comparative study.

#### 2.3.2 Surface Wettability

Water contact angles (WCAs) were measured using the sessile drop method and KRUSS DSA25S equipment (Germany) to evaluate surface hydrophilicity and wettability features. The measurement was carried out while keeping a constant temperature of 20 °C and using a water drop volume of 5 μL to ensure precision. Four repetitions of each WCA measurement were performed, and the average result was considered. Before measuring, every sample was washed with MQ water and dried in vacuum for 24 h at 25 °C.

#### 2.3.3 Membrane Morphology

A Carl Zeiss Neon40 CrossbeamTM field emission scanning electron microscopy (FESEM) instrument, running at a 5 kV acceleration voltage, was used to perform cross-sectional and surface imaging. To preserve their structural integrity, the membrane samples were carefully divided into sections using a scalpel before being subjected to a FESEM examination. The samples were then coated using an ion sputter coater, LITH-SD900C (USA), with a carbon layer that was 5 nm thick.

#### 2.3.4 Surface Roughness

For atomic force microscopy (AFM), a VEECO Dimension 3000 microscope was used in tapping mode to measure the surface roughness and topography of the membranes. In ambient conditions, the device was used in tapping mode to record the phase and topographical images simultaneously. Depending on how the sample surface and tip interact, AFM can provide information with a resolution below a nanometer regarding the topography, magnetic structure, electric charge distribution, material contrast, work function, etc. Following the nanofiltration framework, it was determined that commercial silicon “soft tapping mode” cantilevers (PPP-NCSTR probes from NanoAndMore GMBH) with an incorporated pyramidal tip featuring a radius of curvature of 125 μm, a 5 N/m nominal spring constant, and a nominal fundamental resonance frequency of 150 kHz were utilized. The root-mean-square (RMS) roughness, derived from the standard deviation of the height measurement data (*Z* values), was computed using (eq 1).^24^

1 $$RMS=\sqrt{\frac{1}{N}\sum_{i=1}^{n}{\left|Z_{i}-\mathrm{Z̅}\right|}^{2}}$$

where *N* is the total number of data points available in the dataset, *n* is the number of data points being considered in calculating RMS, Z̅ is the *Z* value’s average in the specified area, and *Z*_*i*_ is the current *Z* value.

#### 2.3.5 PEMM Thickness

The alpha-SE (J.A. Woollam), a single-wavelength variable angle ellipsometry SW VAE, was utilized to measure the thickness of deposited multilayers directly on a raw membrane active surface. Spectroscopic ellipsometry (SE) is a non-destructive technique that measures optical properties through changes in the polarization state of reflected light, represented by psi (Ψ) and delta (Δ). These parameters, linked to the reflectance ratio (ρ) via reflection coefficients *r*_p_ and *r*_s_ (eq 2), help build an optical model, enabling the iterative fitting process for accurate estimation of film thickness and refractive index.^25^

The thickness measurements were repeated two times on three different areas for each modified membrane; the optimal measurements were selected with the lowest error, typically ranging from 3 to 8 mean square error (MSE). Measurements were done at incident angles of 70° and 75° and covering a spectral wavelength range from 280 to 900 nm (spanning from ultraviolet to near-infrared) with a 5 nm step; the SE system captured spectra of picoseconds per square inch (Ψ) and pixel spectra (Δ) across the wavelength (λ) spectrum. Applying the focus probes helped to reduce the spot size. The STBaseline software facilitated parameter fitting within dispersion models, ensuring that calculated Ψ and Δ closely matched measured values, thus enabling extraction of complex refractive indices and film thickness.^26^ While prior studies primarily focused on silicon wafer analysis, this work necessitated modeling for multilayer thickness, thus adopting a tailored approach.^27–30^ The optimal combination of models, minimizing errors, comprised the Cauchy model (eq 3,4) for the raw membrane as a substrate and the Sellmeier advanced model (eq 5) for the modified film. The Sellmeier dispersion model, widely employed for delineating refractive index spectral dependencies within the transparent region, assumes an absence of the extinction coefficient (*k*), simplifying calculations. Conversely, the Cauchy model, integrating exponential absorption tailing, proved essential in accounting for sub-bandgap absorption, particularly near the fundamental absorption edge (∼3.3 eV), where excitonic effects play a pivotal role.^31^

2 $$\rho =\frac{r_{p}}{r_{s}}=\tan\left(\Psi \right)\;e^{i\Delta }$$

3 $$n\left(\lambda \right)=A+\frac{B}{\lambda^{2}}+\frac{C}{\lambda^{4}}$$

4 $$k\left(\lambda \right)=\alpha \;\exp\left\{\beta \left[12,400\left(\frac{1}{\lambda }-\frac{1}{\gamma }\right)\right]\right\}$$

5 $$n^{2}\left(\lambda \right)=1+\frac{A\lambda^{2}}{\left(\lambda^{2}-B^{2}\right)}$$

#### 2.3.6 Zeta Potential

The zeta potential (ζ) of the bare membrane and modified membranes with the highest number of bilayers (6.5 BLs) and their duplicate was measured using the SurPASS 3 (Anton Paar) via streaming potential and current methods. Samples (2 × 1 cm^2^) formed a channel (80–150 μm gap) for 10 mM KCl electrolyte flow, to determine the isoelectric point (IEP). Each membrane underwent three rinses and two measurement cycles for accuracy.

The zeta potential (ζ) value was calculated through the equipment software using the Smoluchowski eq 6.^32^

6 $$\zeta =\frac{dU}{dp}\frac{\eta }{\varepsilon \varepsilon_{0}}k$$

In eq 6, *U* represents the streaming potential, *p* the pressure, η the electrolyte solution’s viscosity, ε the dielectric constant, ε_0_ the vacuum permittivity, and *k* the electrolyte conductivity.

#### 2.3.7 Raman Image Acquisition

Raman spectral imaging was used to analyze the membrane’s cross-section and top surface. Three series of cross-section images were obtained from manually cut samples (with scalp): one from the unmodified (raw) and two from modified membranes (PAH/PSS)­6.5 and (PDADMAC/PSS)­6.5. Additionally, top surface images were captured from raw and modified membranes with 1.5, 4.5, and 6.5 bilayers (BLs) for both PAH and PDADMAC modifications.

A Renishaw inViaTM Qontor Confocal Raman microscope (Renishaw Ltd., UK) was used for spectral image collection. A 785 nm laser was operated at a 50% power (equal to 150 mW) and focused through a 20× objective lens. Spectra were recorded on a Peltier-cooled CCD array detector using point mapping with an exposure time of 0.1 s per pixel. The spectral range for cross-section images spanned from 614.98 to 1722.32 cm^–1^, while for top surface images, it ranged from 390.66 to 1542.08 cm^–1^. The average spectral resolution was approximately 1 cm^–1^. The pixel size was set to 1 × 1 μm^2^. For analysis, a total approximate sample area of 4400 μm^2^ was used for cross-section images, whereas for top surfaces, the analyzed area was approximately 6500 μm^2^. Prior to data analysis, the spectral image underwent preprocessing steps to remove cosmic spikes, reduce noise, and correct for baseline variations.

#### 2.3.8 Chemometric Analysis of Spectral Images

Multivariate curve resolution-alternating least squares (MCR-ALS) was employed to analyze the spectral imaging data obtained from the membrane samples. This chemometric method was chosen for its ability to decompose the complex raw image spectral information into individual pure component contributions, expressed by the related concentration maps and spectral signatures, without prior knowledge about the pure component spectra^33^

The spectral imaging data, initially in the form of a three-dimensional array (*x*, *y*, λ), where *x* and *y* represent the number of pixels in the two spatial dimensions and λ is the number of channels in the spectral dimension, was transformed into a two-dimensional matrix *D*. This transformation was achieved by unfolding the pixel spectra in the original image cube into a data matrix *D*, where each row corresponds to a pixel in the image, and each column represents a specific spectral channel. The dimensions of *D* are (*x* × *y*) rows by λ columns.

The MCR-ALS algorithm decomposes data matrix *D* into two matrices: *C*, representing the concentration profiles of the resolved components, and *S* ^T^, containing their corresponding pure spectra. This decomposition can be expressed according to the bilinear model as^34,35^

7 $$D=CS^{T}+E$$

where *E* contains the variance not explained by the bilinear model, related to the experimental error. The algorithm iteratively optimizes *C* and *S* ^T^ matrices to minimize *E*, subject to chemically meaningful constraints, such as non-negativity. After the MCR-ALS resolution of the spectral image, pure distribution maps of the image constituents can be obtained by folding back the stretched concentration profiles in *C* to match the original 2D spatial structure of the image (see Figure 2).

**Figure 2:**
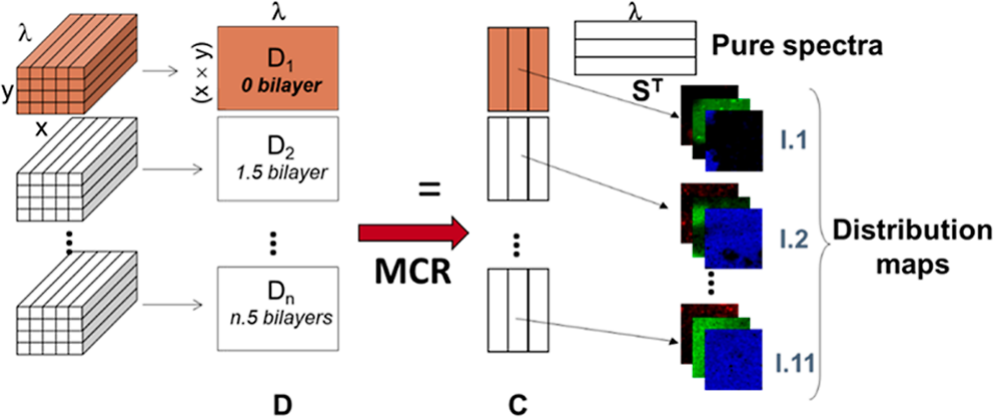
MCR-ALS analysis of an image multiset, where *x* and *y* are spatial pixels and λ represents the spectra wavelengths.

Figure 2 shows an example of an MCR-ALS analysis of an image multiset formed by an ensemble of related images that are analyzed simultaneously. The bilinear model of MCR-ALS also holds for image multiset analysis.^35,36^ In this case, a multiset structure *D* is built by appending the submatrices *D*_n_ linked to the pixel spectra of the images collected from different samples using the same spectroscopic technique.

The decomposition of the multiset structure using (eq 6) provides a single matrix *S* ^T^ of pure spectra, valid for all of the images analyzed, and a matrix *C*, formed by as many *C*_i_ submatrices as images in the data set. The profiles in each of these *C*_i_ submatrices can be appropriately folded back to recover the related distribution maps of the images recorded for each sample (see Figure 2). The MCR-ALS analysis was carried out using the MCR-ALS GUI 2.0 interface developed under MATLAB and related in-house coded routines.^37^

### 2.4 Membrane Performance

The process flow diagram illustrates the experimental setup for NF in a closed-loop system (Figure 3). The setup includes a GE SEPA CF II cross-flow membrane cell with an effective membrane area of 140 cm^2^ and a Hydra-Cell diaphragm pump, which circulates a 10 L feed solution maintained at 25 ± 3 °C. The feed stream, shown in red, enters the membrane system, while the permeate, represented in blue, is the filtered solution collected for analysis. Both the retentate (represented in black) and permeate are recirculated back to the feed tank, ensuring a continuous closed-loop operation. To regulate the process, transmembrane pressure (TMP) and cross-flow velocity (CFV) were adjusted using a needle valve and a bypass valve. Pressure, temperature, and flow rate were continuously monitored through sensors, including pressure transmitters (PIT), temperature indicator transmitters (TIT), and flow meter (QIT). The system design ensures stable and consistent membrane performance by allowing continuous recirculation of the solution, preventing rapid concentration polarization, and ensuring uniform testing conditions.

**Figure 3:**
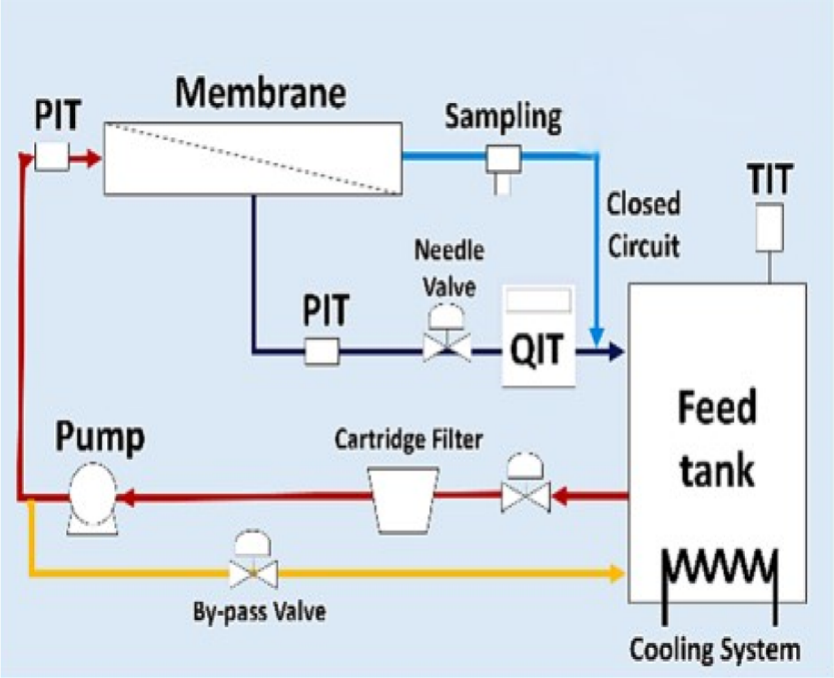
The process flow diagram of the NF setup. Copyright 2024 Elsevier.^38^

Membranes were presoaked in Milli-Q water overnight, assembled with spacers, and pressurized with deionized water at 20 bar (5.04 ± 0.1 L/min, CFV = 1 m/s) before the feed solution was filtered by NF. The feed solution was prepared as a mixture of NaCl (400 mg/L), CaSO_4_ (300 mg/L), and MgSO_4_ (100 mg/L) with a pH of 8–9. For the NF tests with the feed solution, the transmembrane pressure (TMP) ranged from osmotic pressure 2 to 20 bar for species rejection analysis. Permeate mass was measured for flux calculations, and the system was cleaned with deionized water at 15 bar (CFV = 1 m/s) after each test. The virgin membrane and all modified membranes, along with their duplicates, were tested to ensure the accuracy of the results. An Agilent Technologies 7800 inductively coupled plasma mass spectrometer and a 5100 inductively coupled plasma optical emission spectrometer were utilized to determine the concentrations of various elements in the collected permeate samples.

## 3 Results and Discussion

### 3.1 Membrane Composition (FTIR Analysis)

The raw semiaromatic polyamide membrane was analyzed using FTIR analysis to determine the materials it contained based on its chemical composition. To the best of the authors’ knowledge, no prior literature exists on the FTIR characterization of the semiaromatic polyamide membrane. It was challenging to perform FTIR analysis on the membrane without knowledge of the materials employed in each layer. The overlap of similar bonds that may be present in different membrane layers was also a significant aspect to be considered when analyzing the spectra of these membranes because each membrane might be made up of various layers, and the beam will pass through all of them. NF membranes generally have the same fundamental structures of support, substrate, and active layer. Any modifications were made to the active layer (top layer). Therefore, it was intended to divide the raw membrane into its top and base halves to reduce overlap and, as a result, increase the likelihood of recognizing the material and alteration. The top layer corresponded to both the active layer and substrate, and the base corresponded to the support layer. The FTIR spectra of the top layer of the raw semiaromatic polyamide membrane (blue) and the base layer (red) are represented in Figure 4a. According to observed bonds in the active layer (generally in the range of 1500–4000 cm^–1^), the structure seemed to be a semiaromatic poly­(piperazine amide), by aliphatic primary amine and alcohol (3300–3400 cm^–1^ and 3200–3550 cm^–1^), methylene (2840–3000 cm^–1^), and amide I (1620 cm^–1^).^39,40^ The substrate was a polysulfone layer by aromatic in-plane ring bend stretching vibration (1586, 1505, 1488 cm^–1^), C–H symmetric deformation of C­(CH_3_)_2_ (1365–1385 cm^–1^), asymmetric SO_2_ stretching vibration (1294–1323 cm^–1^), C–O–C asymmetric stretching vibration of the aryl-*O*-aryl group (1241 cm^–1^), symmetric SO_2_ stretching vibration (1151–1170 cm^–1^), and in-phase out-of-plane hydrogen deformation of a para-substituted phenyl group (833 cm^–1^).^41^ The appeared peaks in the support layer corresponded to the polyethylene terephthalate (PET) because of carbonyl stretching (1711 cm^–1^), carbon monoxide stretching (1341, 1410 cm^–1^), deformation of the terephthalate residues (1242 cm^–1^), methylene group (1094 cm^–1^), and aromatic ring (846, 871, 970 cm^–1^).^42^ So, based on FTIR observations, the semiaromatic polyamide membrane was a nanofiltration membrane with a PET support, a polysulfone substrate, and a semi-aromatic poly­(piperazine amide) active layer (see Figure 4a).

**Figure 4:**
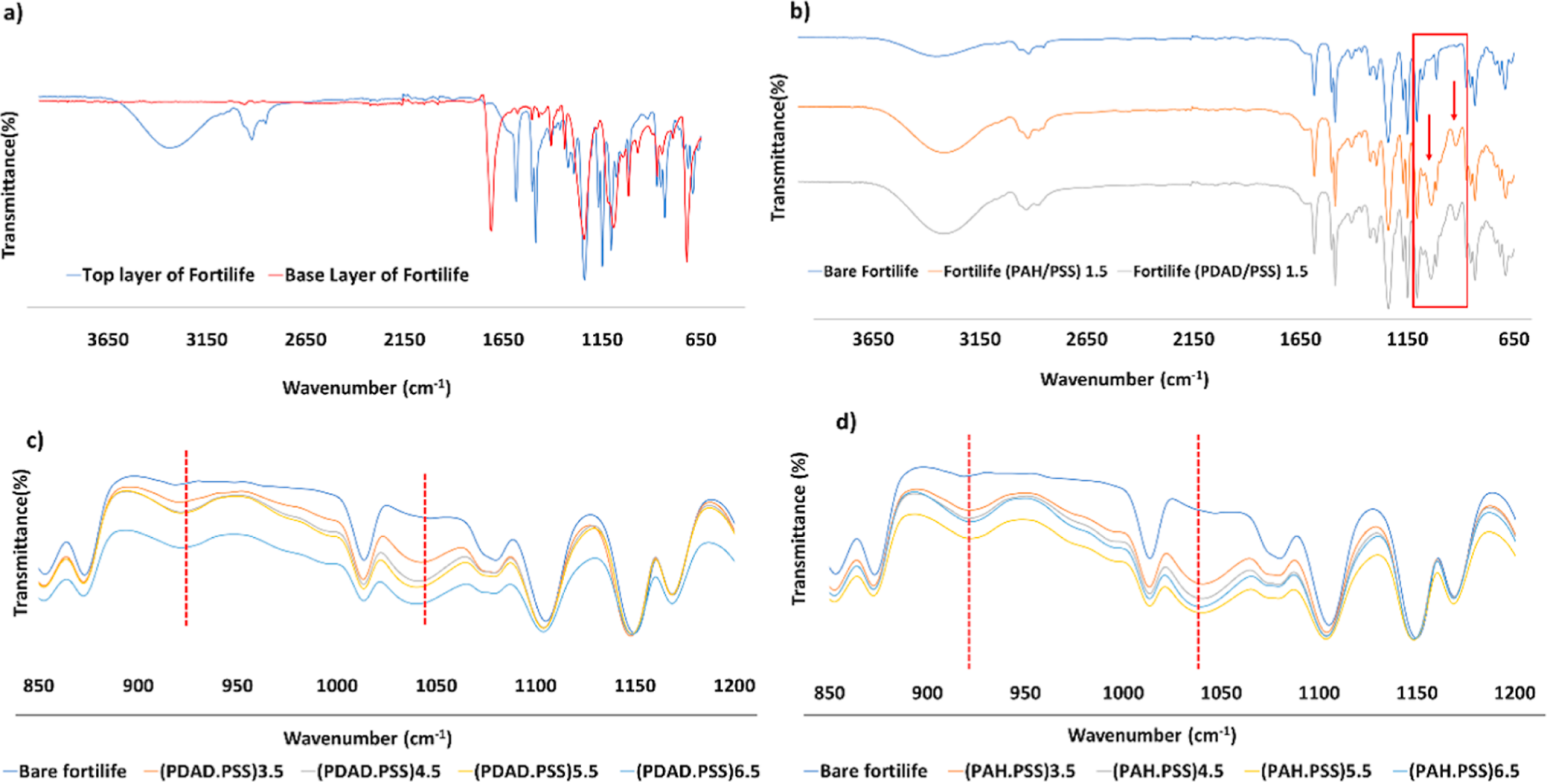
FTIR spectra of (a) the raw Fortilife membrane and (b) the modified membranes with 1.5 BLs of PAH (orange) and PDADMAC (gray), compared to the bare membrane (blue). The changes in intensity of new peaks in modified membranes of (c) (PDADMAC/PSS) (3.5–6.5 BLs) and (d) (PAH/PSS) (3.5–6.5 BLs).

After deposition of 1.5 BLs, there were two additional peaks at 1039 and 922 cm^–1^ in the spectra of coated semiaromatic polyamide compared to the raw membrane’s spectra (see Figure 4b). The peak at 1039 cm^–1^ is attributed to the sulfonate stretching of PSS and the C–N stretching of PDADMAC or PAH, confirming the successful coating of 1.5 layers. All modified membranes with (PDADMAC/PSS) and (PAH/PSS) bilayers from 1.5 to 6.5 bilayers (BLs) showed similar additional peaks. To compare the intensities of these new peaks as the number of layers increased (from 3.5 to 6.5 BLs), the spectral data of the modified membranes were normalized (see Figure 4c,d). As expected, the results showed that the intensity of these peaks grew with the number of layers, indicating a greater presence of the modified materials in both PDADMAC/PSS and PAH/PSS. This observed trend aligns with findings for PDADMAC/PSS reported in other studies as well.^10,43,44^

### 3.2 Membrane Surface Morphology and Topography

The effect of the number of bilayers on the contact angles (CAs) of coated semiaromatic polyamide membranes was measured by the sessile drop method. The contact angle (CA) of the semiaromatic polyamide membrane was measured at 30° ± 1, consistent with values reported for similar nanofiltration (NF) membranes, including 33.7° for NF270, 44.6° for DL, 36° for VNF1, and 30° for NFX membranes.^10,45^ The active surface of nanofiltration membranes is typically made of hydrophilic materials such as polyamide, sulfonated poly­(ether sulfone), and sulfamide, meaning they have a strong affinity for water.^46,47^

The contact angle increased to 42° with (PDADMAC/PSS)­1.5 modification and was even more hydrophobic with (PAH/PSS)­1.5 modification to 61°. The CA of coated membranes was higher than that of raw membranes due to the hydrophobic nature of the PDADMAC and PAH polyelectrolytes (Supporting Information Sections D and F). The CA for both series of modified membranes sharply increased until 3.5 bilayers (see Figure 5). Consequently, it is advisable to start the modification process with a higher number of layers (4.5) to attain superior modification in terms of water affinity. Modifying membranes altered their overall properties, depending on the final terminating layer. This effect is known as the odd–even effect. The odd–even effect refers to the difference in material properties based on whether the number of layers is odd or even. In LBL modification, membranes with different numbers of layers often show distinct properties due to the influence of the final terminating layer.^48^ Moradi et al. and de Grooth et al. reported that membranes with polycation terminations, such as PDADMAC and PAH, show reduced hydrophilicity in the initial modified layers due to the properties of these polycations. However, hydrophilicity increases as more layers are added.^10,48^

**Figure 5:**
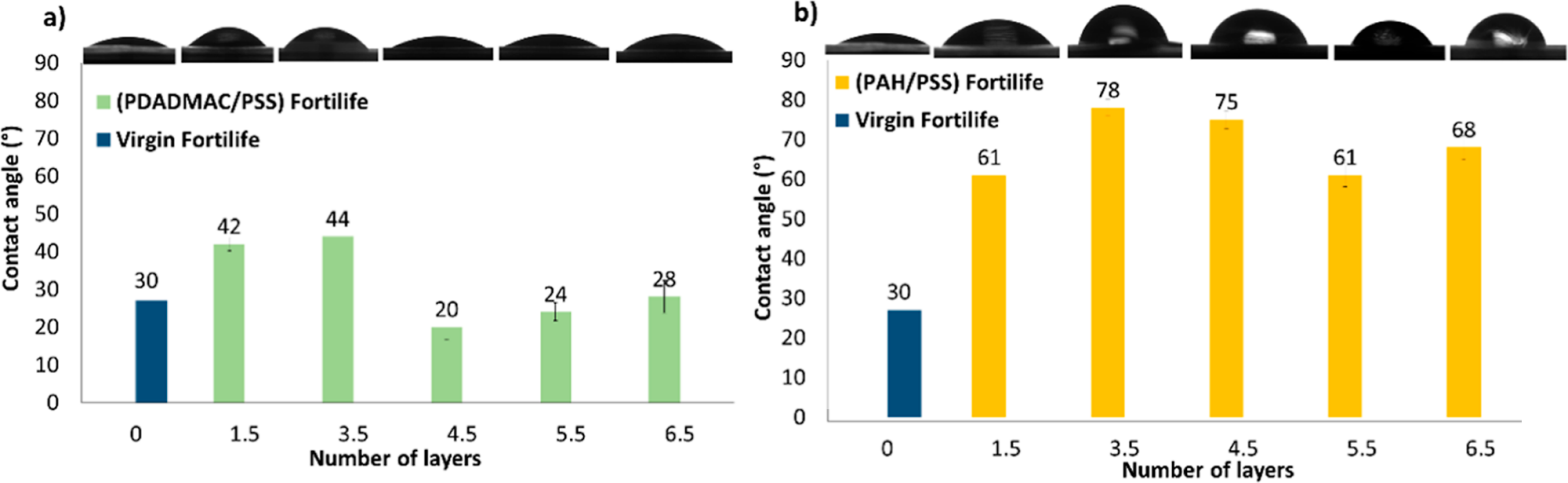
The contact angle of (a) (PDADMAC/PSS) (1.5–6.5 BLs) (green) and (b) (PAH/PSS) (1.5–6.5 BLs) (yellow) in comparison to the raw Fortilife membrane (blue).

A significant distinction was evident between the two distinct polycations utilized: After 3.5 bilayers of PDADMAC, the membrane’s hydrophilicity became similar to that of the raw membrane. Adding more layers up to 6.5 bilayers maintained this hydrophilic state, with the contact angle consistently ranging between 20° and 30° (Figure 5a). But in the case of PAH, even with 6.5 bi-layers, the hydrophilicity remained substantially lower than that of the raw membrane (see Figure 5b). This discrepancy may impact the duration and pressure required for solution passage through the membrane, resulting in heightened energy consumption and operational costs.^49,50^ Notably, the modification with (PDADMAC/PSS) was anticipated to elicit a more substantial decrease in contact angle relative to (PAH/PSS), attributable to the heightened positive charge density associated with PDADMAC. According to Ouyang et al., PDADMAC has stronger capillary effects and swells more than PAH due to its higher ionic strength, leading to a more significant increase in hydrophilicity with additional layers.^51^

Elzbieciak et al.^29^ studied the changes in CA of LBL-modified membranes at different pH and their documentation of average contact angle variations at a pH range around 7 harmonized with measurements conducted within PDADMAC (30°) and PAH (60°) in this study. Various studies have indicated that PDADMAC’s contact angle is pH-independent, unlike the pH-dependent behavior of PAH. In PAH/PSS multilayers, reduced PAH dissociation under basic conditions, due to NH_3_(I)­group deprotonation, decreases the number of charged groups along the polymer chain.^19,28,52^

Based on the images captured through FE-SEM in Figure 6, the structure of the raw semiaromatic membrane was examined from two perspectives: surface views (see Figure 6a–c) and cross-sectional views (see Figure 6d). Figure 6a, magnified at 6 K, shows that the pristine membrane had a smooth, uniformly distributed active surface. Image (b) shows the free volume within the polysulfone matrix. Image (c), focused on the support surface, shows filament-like structures resembling PET fibers that were consistent with previous FTIR identifications (Section 3.1). Particularly, in the cross-section (see Figure 6d) (d1), the PET support structure was discernible, displaying an asymmetrical porous configuration measuring 98 ± 1 μm in thickness. Furthermore, the polysulfone substrate exhibited a densely porous structure (d2) with a thickness of 64 ± 1 μm, while the active surface appeared extremely thin and uniform at the nanometer scale (d3). The membrane’s total thickness was estimated to be approximately 162 ± 2 μm.

**Figure 6:**
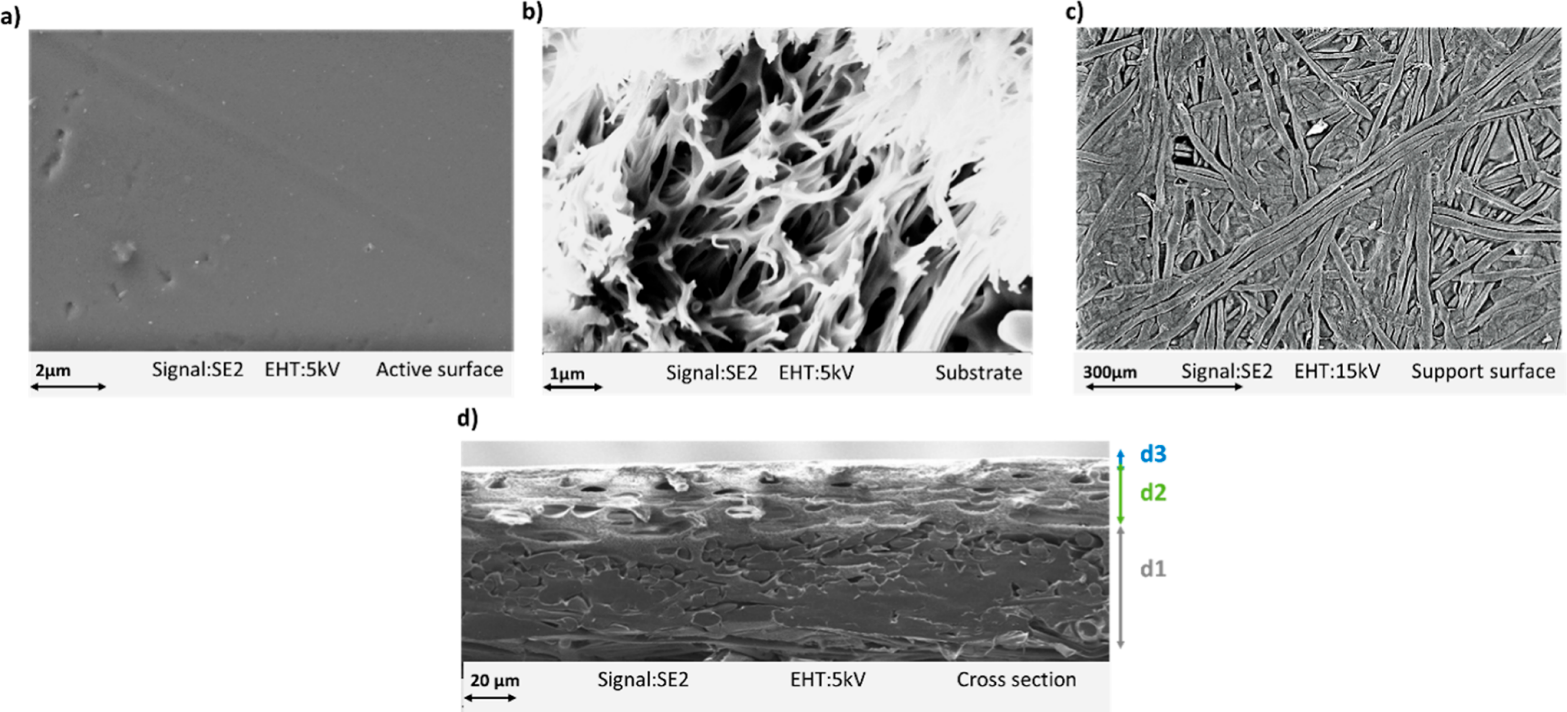
FE-SEM images of raw Fortilife membrane’s (a) active surface layer, (b) substrate surface layer, (c) support surface layer, and (d) cross-section including support (d1), substrate (d2), and active layer (d3).

The deposition of polyelectrolytes onto the active layer was evident in the (PDADMAC/PSS) and (PAH/PSS) modifications, even with just 1.5 BLs, indicating the successful implementation of the modification process (see Figure S2a in the Supporting Information). Due to the stronger interactions between PDADMAC and the membrane surface, the (PDADMAC/PSS) modification resulted in a smoother surface morphology than PAH/PSS. However, as the number of layers increased, the surface morphology transformed into a bulkier structure, characterized by the emergence of hills and polymer filaments instead of particles. This transformation undoubtedly impacted surface roughness. Complementary analyses of surface roughness and heterogeneity were needed, alongside FE-SEM imaging, to better understand and optimize modification uniformity and morphology.

AFM analysis was performed on both raw and modified membranes. For each membrane, two different samples were measured at two distinct sites within a 20 × 20 μm^2^ area to enhance precision. The raw membrane showed a very smooth surface with an average root-mean-square (RMS) roughness of 11 nm. For both groups of modified membranes, increasing the number of layers led to a rougher active surface. Based on FE-SEM images, it was expected that the PAH-coated membranes would have a rougher surface compared to the PDADMAC-coated membranes, which was confirmed through AFM analysis (see Figure 7).

**Figure 7:**
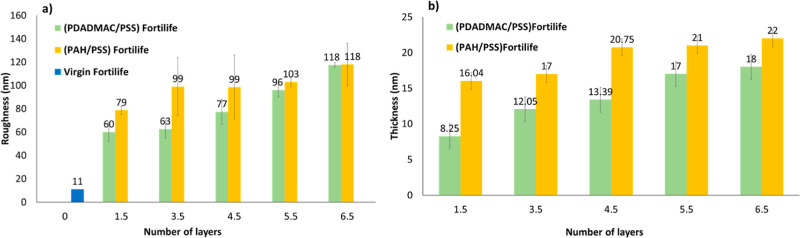
Comparison of (a) active surface roughness and (b) multilayer thickness between raw (blue) and modified membranes (1.5–6.5 BL) for PDADMAC/PSS (green) and PAH/PSS (yellow).

PDADMAC/PSS formed smoother and more compact layers than the PAH/PSS membranes. The smoothness of both PDADMAC/PSS and PAH/PSS multilayers can be influenced by factors such as ionic strength and deposition conditions, including pH (detailed in Supporting Information D–F). Compared to PAH, PDADMAC is a stronger polyelectrolyte with a consistent charge and higher ionic strength, which enhances electrostatic screening, improves polymer chain interpenetration, and leads to smoother layers.^53,54^ Zan et al. reported that fully charged PDADMAC and PSS form noticeably thin layers. Under the applied salt concentration (50 mM NaCl), these polyelectrolytes are expected to adsorb in a flat arrangement, leading to the formation of compact layers.^55^

The RMS results often correlate with the surface wettability. A similar trend in surface roughness (see Figure 7) and CA was observed for both polycations. CA and surface roughness sharply increased up to 3.5 bilayers, after which changes in the statistical height roughness became minimal. Mu et al. also confirmed that the higher surface roughness corresponds to higher contact angles, suggesting the surface is less wettable.^11^ The surface energy, capillary action, surface heterogeneity, and chemical composition are some elements that affect the link between contact angle and surface roughness.^56^ For (PDADMAC/PSS) (4.5–6.5), the roughness of the surface effectively increased the surface area and caused the CA to decrease (in contrast with the general correlation between surface CA and roughness mentioned before); however, it fitted with other published studies. For example, Moradi et al. reported an increase in surface roughness for (PDADMAC/PSS)­5.5, from 9.9 to 15 nm in comparison with the DL raw NF membrane, which enhanced water uptake due to larger surface area interactions.^10^ In the case of PAH, by increasing the number of layers (4.5–6.5), still a significantly higher surface roughness (99–118 nm) than the raw membrane (11 nm) was observed (see Figure 7a) and the same behavior for surface CA was observed (see Figure 5b). It is noteworthy to highlight that for PAH/PSS at 3.5, 4.5, and 6.5 bilayers, the standard deviations were around 20 nm. This phenomenon could be related to the existence of elevated areas and a substantial structure, resulting in fluctuations in roughness across various parts of the sample.

Based on the achieved results, (PDADMAC/PSS)­4.5 with a CA of 20° and a roughness of 77 nm and (PAH/PSS)­1.5 with a CA of 61° and a roughness of 79 nm, because of their better combination of wettability and roughness, among other modified membranes, may help minimize the membrane’s fouling.^49,50^ Fouling occurs when impurities or particles adhere to the membrane surface, gradually reducing performance.^11^ Smoother surfaces with lower contact angles have better wettability, making it easier to remove adhesive materials and reducing the likelihood of fouling. This also enhances selectivity, particularly during separation processes.^49^ A smoother surface minimizes the chances of flaws or irregularities, preventing undesirable molecules or ions from passing through the membrane and compromising its selectivity.^11^

Ellipsometry measurements of the deposited multilayer thickness on the raw membrane’s active surface showed that the thickness increased with the number of layers. (PDADMAC/PSS) exhibited a lower thickness compared to PAH/PSS (see Figure 7b), which aligns with the AFM results (see Figure 7a). The thickness varied from 8.3 to 18.0 ± 0.1 nm for (PDADMAC/PSS) and from 16.1 to 22.0 ± 0.1 nm for (PAH/PSS) as the number of layers increased from 1.5 to 6.5. In the state of the art, most of the ellipsometry measurements have been done on the modified membranes, resulting in a bi-layer, and approximately each bi-layer gives an enhancement of roughly 1–2 nm.^28,57^ No prior studies utilize the ellipsometry method and modeling specifically aimed at measuring the thickness of the PEMMs directly on the raw semiaromatic membrane. To accurately measure the modified film thickness, an advanced Sellmeier model was utilized, which yielded the lowest error. It is noteworthy that previous studies employed the Cauchy model for measuring modified films on silicon wafers.^27,28^ However, since the objective of this work was to measure the thickness of the modified layer directly on bare membranes, it was discovered that by combining the Cauchy model for the substrate with the Sellmeier model for the modified layer, more precise results were achieved. While the Cauchy model is widely used for its simplicity, it may not adequately characterize thin, diverse layers like PEMMs. Conversely, the Sellmeier model provides enhanced flexibility and accuracy, particularly for materials with complex optical properties and surfaces of varying roughness.^26^ This made it a better choice for ellipsometry measurements of thin PEMM layers. The thickness of PEMMs significantly influences properties such as permeability, selectivity, mechanical strength, and surface charge. Measuring thickness allows for optimizing deposition parameters, providing insights into the internal structure and how deposition parameters affect morphology, porosity, and density.^25^ Accurate thickness measurement serves as quality control, ensuring reproducibility and uniformity in fabrication and predicting membrane performance. The thin polymer deposition layers make measuring thickness challenging in surface modification.

Zeta potential measurements showed that the isoelectric point (IEP) for the unmodified membrane was 2.7, while the PDADMAC-modified membrane reached an IEP of 4.9, and the PAH-modified membrane had an IEP of 3.4 at the highest bilayer count (6.5 BLs). The zeta potential results of the bare membrane confirm the membrane’s expected negative charge, consistent with its composition (see Figure 4a). Amine protonation causes slight positivity at low pH, while carboxyl deprotonation leads to strong negativity above the IEP (2.7). The modified membranes approached neutrality or their respective IEPs more quickly when the pH was decreased, and protons were introduced during the surface modification process. The higher IEP of PDADMAC-modified membranes compared to PAH can be attributed to differences in ionic strength and chemical composition.^58^ PDADMAC, a quaternary ammonium polyelectrolyte, maintains a permanent positive charge, ensuring robust cationic properties across a wide pH range. In contrast, PAH, with its primary amine groups, gradually loses its charge as the pH increases, resulting in a lower IEP.

### 3.3 Chemometric Analysis for Distribution and Uniformity of Membrane Modification Using Layer-by-Layer Deposition

#### 3.3.1 Cross-Section Raman Images

Cross-section images were taken of both the raw membrane and the modified membranes with the highest number of layers (6.5), as modifications are more noticeable in samples with greater thickness. Spectra were collected at various points on the substrate and the support layer of both raw and modified membranes. In all membrane samples, regardless of treatment, common materials were identified. Figure S3 in the Supporting Information illustrates the spectra from the substrate area in red and those from the support area in blue of the raw membrane. The findings from Raman analysis about membrane composition align with the FTIR results (discussed in Section 3.1). The red spectra showed characteristic PS and PA bands at 800 cm^–1^ (out-of-plane CH of benzene), 1050–1200 cm^–1^ (asymmetric C–O–C stretching and C–H in-plane of benzene and ring-breathing coupled with C–S and C–O stretching).^59^ PA was identified with ∼1634 cm^–1^ (amide I band of polyamide) and minor bands at 1213 cm^–1^ (N–H wagging).^60^ The blue spectra displayed characteristic PET bands: around 600 cm^–1^ (C–C–C in-plane bending), 850 cm^–1^ (benzene ring vibration), and a strong band at 1600 cm^–1^ (C=C stretching of the benzene ring).^61^

To analyze the membrane composition and identify modifications using MCR-ALS, two image multisets were examined for cross-section measurements. One multiset consisted of the Raman images of the bare membrane and the (PAH/PSS)­6.5-modified membrane, while the other consisted of the bare membrane and the (PDADMAC/PSS)­6.5-modified membrane. Each MCR-ALS analysis was limited to three components, applying non-negativity constraints on both concentration and spectral profiles. Including additional components in the MCR-ALS analysis did not improve the results. Both multisets could be described with three components with the same spectral identity. For simplicity, Figure 8a presents the MCR-ALS distribution maps of the virgin Fortilife membrane and (PAH/PSS)­6.5-modified membrane from the first multiset analysis and only the maps of (PDADMAC/PSS)­6.5 from the second multiset analysis. Figure 8b shows the pure spectra for the three membrane components found (very similar in the two multiset analyses carried out). Based on the peaks identified in Figure S3 in the Supporting Information, compound A corresponds to the support layer (PET), compound B corresponds to the substrate (polysulfone), and compound C corresponds to the modification layer. The distribution maps in Figure 8a match the morphology expected in the cross sections analyzed. The component in the B map (substrate) is always on top of the membrane, whereas compound A (support layer) is at the bottom. In the maps of the modified membranes, compound C (related to the modification layer on the surface of modified membranes with 6.5 BLs) shows up as a light cyan line above the substrate layer and is not present on the bare membrane. As expected, this modified bilayer coating is significantly thinner than the bare membrane materials.

**Figure 8:**
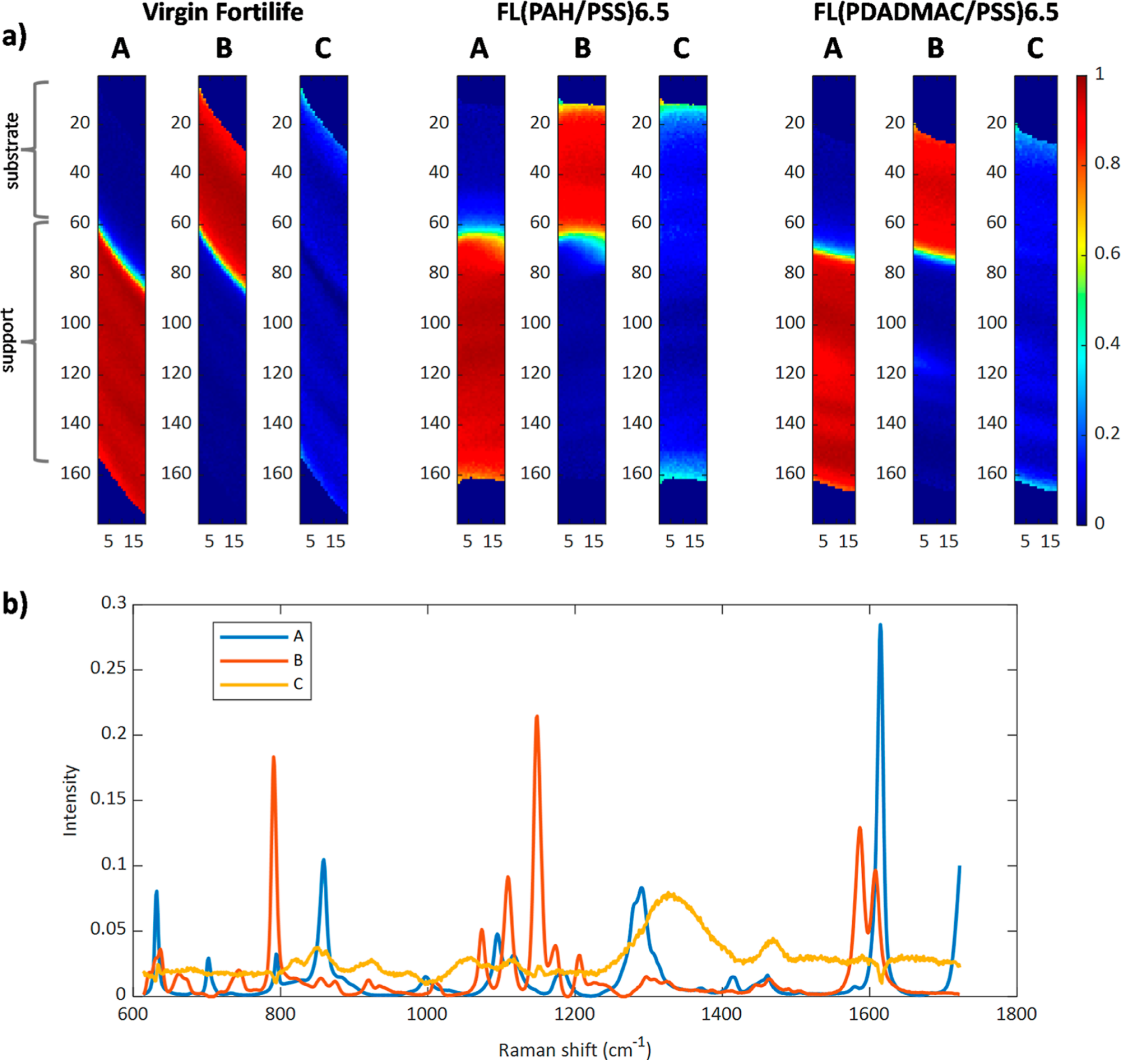
(a) Concentration maps of raw Fortilife, FL­(PAH/PSS)­6.5, and FL­(PDADMAC/PSS)­6.5. (b) Pure spectra for all membranes. A (support, PET), B (substrate, polysulfone), and C (modification, PAH or PDADMAC).

#### 3.3.2 Analysis of the Top-Surface Images of Layer-by-Layer Modified Membranes

The top surface of modified membranes with different numbers of bilayers (1.5, 4.5, and 6.5) for both polyelectrolytes (PAH and PDADMAC) was scanned in an area covering around 6500 μm^2^. Figure S4 in the Supporting Information provides an initial comparison of the average spectra for membranes with the highest number of modification bilayers (6.5 BLs) for each polycation (PAH in red and PDADMAC in blue) and the unmodified membrane (in black). The main observation is the clear spectral differences between the untreated and modified membranes, particularly in the ranges of 400–600 cm^–1^, 800–1000 cm^–1^, and around 1450 cm^–1^. Additionally, there was no spectroscopic distinction between the modifications made with PAH and PDADMAC.

To have a better understanding of the identity and distribution of components on the membrane surfaces, two multiset MCR-ALS were analyzed: one for the series of PAH-modified membranes and another for PDADMAC-modified membranes, with both multisets also including the untreated membrane. The applied constraints included non-negativity for both concentration and pure spectral profiles, as well as correspondence of species,^34,35^ ensuring that the bare membrane did not contain any components related to the modification.

As can be seen in Figure 9b, component (B) corresponds to pure spectra of the bare membrane, where the observed bands were remarkably similar to the substrate component spectra found in the cross-section images (see Figure 8b). This is the membrane material expected to be identified since images were collected confocally on the top of the surface, where the substrate and the modification were contributing to the signal and not the deepest support membrane material. The distribution map of the substrate material showed a negative concentration gradient of the bare membrane component (B) as the number of bilayers increased; i.e., the color turned more yellow, indicating a decrease in the contribution of the raw membrane component to the signal measured (see Figure 9a).

**Figure 9:**
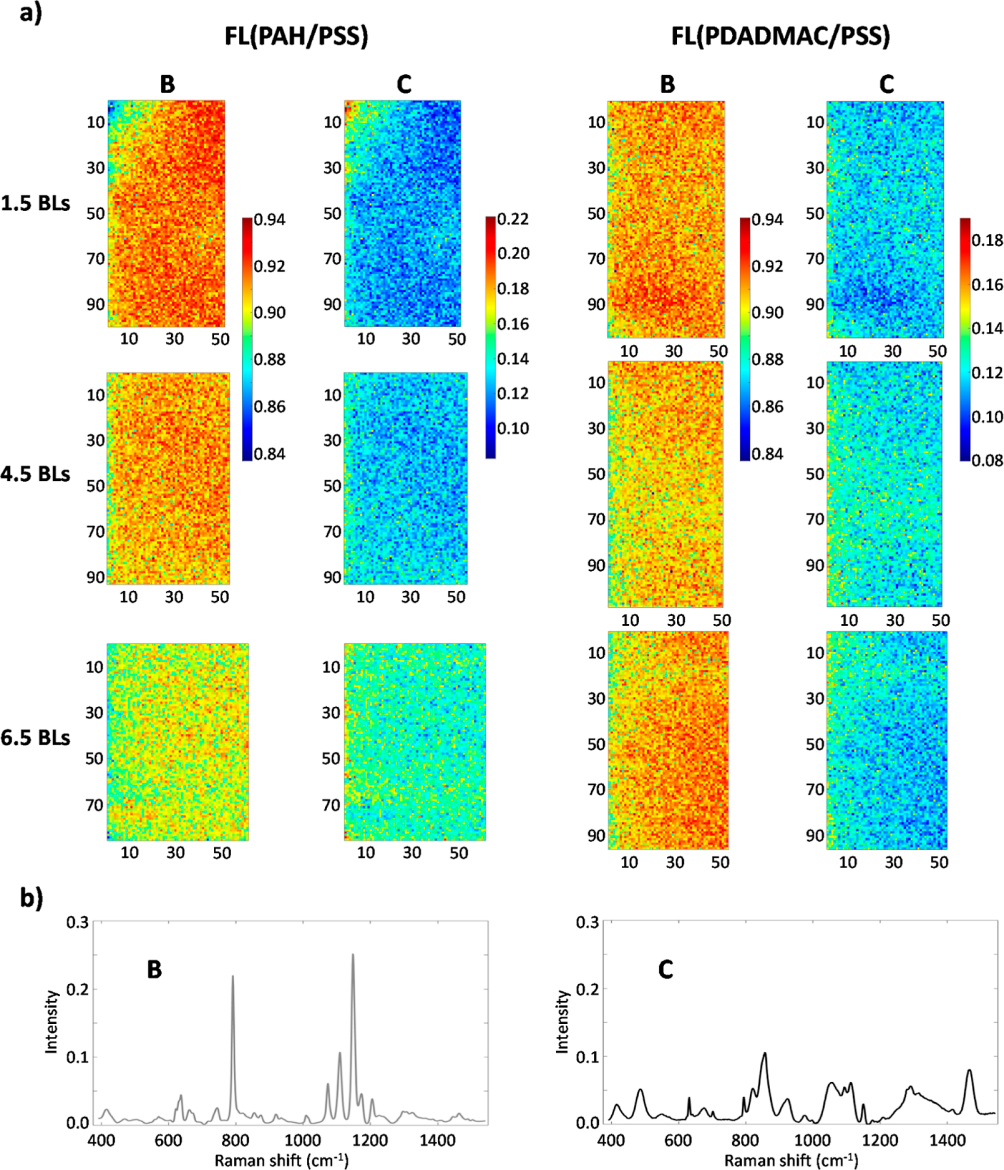
(a) Concentration maps for FL­(PAH/PSS) (1.5, 4.5, and 6.5) membranes and FL (PDADMAC/PSS) (1.5, 4.5, and 6.5) and (b) pure spectra for the substrate layer (B) and the modification (C).

In Figure 9b, component (C) corresponds to the modified membrane layer, and similar bands to the modification spectra in Figure 8b around 800 cm^–1^ and 1200–1450 cm^–1^ can be found in the cross-section images. Moreover, the dominant bands of (C) appeared in the zones where there were more visible differences among the average spectra of the bare membrane and the modified membrane in Figure S4 Supporting Information (those mentioned around 1000 cm^–1^).

The distribution map showed a positive concentration gradient of the modification component (C) as the number of bilayers increased (see Figure 9a). Thus, upon increasing the number of bilayers, the intensity of the component (C) on the surface increased, so the color of the surface changed from bluish to greener, which confirms the increment of the modified material on the active surface. The distribution of the component related to the modified bilayers on the surface is more visible for PAH-modified membranes (greener), which can be related to the higher roughness of these membranes (see Figure 7a) or to the better distribution of polyelectrolytes on the surface in comparison to PDADMAC-modified membranes.

### 3.4 Preliminary NF Testing Results

All membranes and their duplicates were initially pressurized with deionized water for 2 h at a maximum pressure of 20 bar by the NF system. Their water permeability and water flux were measured during the water pressurization process. Following this, the membranes were pressurized and tested with the synthetic solution including NaCl, MgSO_4_, and CaSO_4_ salts at varying pressures ranging from osmotic pressure to 20 bar.

PDAD- and PAH-modified membranes showed different water permeability and water flux in comparison with the bare membrane due to variations in active layer properties, morphology, and hydrophilicity, as mentioned in the Membrane Characterization results section. The bare membrane had a permeability of 9 ± 0.5 L/(m^2^ h bar). The PDAD-modified membranes maintained a similar permeability, as expected based on the contact angle results (see Figure 5). The (PDADMAC/PSS)­6.5 showed the lowest water permeability (8 ± 0.6 L/(m^2^ h bar)) due to increased thickness and roughness (see Figure 7). PAH-modified membranes with 5.5 BLs exhibited the lowest water permeability of 5.4 ± 0.2 L/(m^2^ h bar). This lower permeability was expected compared to the PDADMAC-modified membranes as the PAH-modified membranes displayed higher contact angle (CA) values (see Figure 5). Another key factor is the polyelectrolyte’s structure, which influences the charge density of the deposited film. PAH, having a higher charge density than PDADMAC, leads to the formation of smaller nanopores, resulting in lower water permeability and enhanced separation performance.^62^ Additionally, PAH’s lower molecular weight allowed shorter polymer chains to penetrate and block membrane pores, further decreasing permeability and flux.^63^

Based on the characterization of the (PDADMAC/PSS)­4.5 membrane, it was concluded before in Section 3.2 that it would demonstrate higher permeability and then was confirmed through NF testing (10 ± 0.1 L/(m^2^ h bar)). This conclusion was drawn from its favorable combination of roughness (77 nm) and the contact angle (20°). The characterization of (PAH/PSS)­1.5 with a surface roughness of 79 nm and a contact angle of 61° also indicated a high permeability. NF membrane tests confirmed this value, with an average of 10.5 ± 0.1 L/(m^2^ h bar) for the (PAH/PSS)­1.5 membrane. The slightly higher permeability of the PAH-modified membrane with 1.5 bilayers in comparison with the bare membrane, despite its greater contact angle and roughness, is due to PAH being a weak polycation and the sulfonate group in PSS enhancing hydrophilicity and promoting water transport.^64^

At a pressure of 20 bar, the bare membrane had an average water flux of 191 ± 3 L/(m^2^ h), while PDAD and PAH with 6.5 BLs showed lower average water fluxes of 166 ± 7 and 151 ± 3 L/(m^2^ h), respectively. PAH/PSS formed denser, thicker, less permeable layers than PDADMAC/PSS, which resulted in reduced flux, justifiable with what was observed through differences in characterization of the active surface in previous sections and similar to what has been reported in the literature.^54^ For instance, de Grooth et al.^22^ reported comparable findings, highlighting that those membranes with a PDADMAC-terminated surface generally exhibit higher water flux than those with a PAH-terminated surface due to their increased swelling ability.

During the NF tests with the feed solution, permeate samples were collected over time, and rejection values for each membrane were measured after testing with a mixed salt solution. The goal with the modified membranes was to achieve higher rejection rates for multivalent cations and lower rates for monovalent cations compared with those measured for the bare semiaromatic polyamide membrane. Higher TMPs improved solute rejection in both bare and modified NF membranes by increasing permeate flux, which diluted solutes and enhanced rejection.^65^ The bare membrane exhibited high sulfate rejection (97%) due to its negatively charged active surface at pH 8–9, following Donnan exclusion, while Mg^2+^ (88%) and Ca^2+^ (79%) had lower rejection rates. Na^+^ had lower rejection (around 48%) in comparison with divalent cations due to the effects of dielectric exclusion, which is influenced by the ions’ hydration and charge density.^66^

PDAD-modified membranes with 5.5 and 6.5 BLs had the highest average rejection value in comparison with bare membrane, although the 5.5 BL membrane performed slightly higher rejection of divalent ions, with Mg^2+^ increasing from 88% to 93% and Ca^2+^ from 79% to 90% and sulfate rejection of 99%, while Na^+^ rejection had just a slight increase (48–50%), which can result in enhanced selectivity. PAH-modified membranes with 6.5 BLs showed even greater divalent ion rejection, with Mg^2+^ rising from 88% to 95% and Ca^2+^ rising from 79% to 92%, while sulfate rejection exceeded 99%. Monovalent ion (Na^+^) rejection also varied, increasing to 51%. Overall, PAH/PSS-modified membranes achieved higher divalent ion rejection than PDADMAC/PSS-modified membranes. PAH-modified membranes exhibited higher ion rejection than PDADMAC-modified ones due to increased roughness, contact angle, and a bulkier active surface, as seen in the Membrane Characterization sections. These factors enhance charge interactions, steric hindrance, and electrostatic repulsion, improving divalent cation rejection.^54^

Modified membranes had a less negative surface charge than the virgin membrane (see zeta potential results), leading to a higher rejection of divalent cations. However, optimizing the number and combination of bilayers is crucial for improving the selectivity. PDADMAC membranes with 4.5 and 6.5 BLs exhibited slightly better selectivity than the bare membrane, as the separation between monovalent and divalent cations remained low. PDADMAC/PSS with 5.5 BLs achieved the highest selectivity for both Na/Ca and Na/Mg (see Figure 10a,b, respectively). Concerning PAH-modified membranes, they showed improved selectivity with increasing bilayers, with PAH 6.5 BLs providing the best Na/Ca and Na/Mg selectivity (see Figure 10c,d, respectively). The reduced negative charge of the active layer enhanced divalent cation rejection via Donnan exclusion while allowing easier monovalent cation transport for electroneutrality. The average selectivity of Na/Ca for the bare membrane was 2.6 ± 0.4, which increased to 5.4 ± 0.5 for (PDADMAC/PSS)­5.5 and to 6.1 ± 1.5 for PAH 6.5. For Na/Mg, the selectivity was 4.5 ± 0.3, rising to 6.7 ± 0.6 for (PDADMAC/PSS)­5.5, and to 10.4 ± 1.9 for PAH 6.5. PAH-modified membranes achieved higher selectivity due to their high charge density from easily protonated amine groups, which strengthens binding with PSS and enhances rejection through dielectric exclusion. In contrast, PDADMAC has a larger, more flexible structure with lower charge density, leading to weaker ionic interactions with PSS and a greater reliance on Donnan exclusion.^54^ Few studies can be found in the literature to compare the results. For instance, Baig et al.^67^ conducted one of the few studies on PAH-modified flat-sheet membranes, demonstrating that depositing 4.5 bilayers of polyelectrolyte multilayers (PEMs) effectively transformed aqueous phase separation (APS) support membranes into nanofiltration membranes, achieving MgCl_2_ retention exceeding 90%. Besides, Moradi et al.^10^ applied a similar LBL modification to DL membranes, using brine as a feed solution, showing that (PDAD/PSS)­5.5 delivered the best performance, enhancing Mg^2+^ and Ca^2+^ rejection up to 96% and improving monovalent/divalent selectivity (>21).

**Figure 10:**
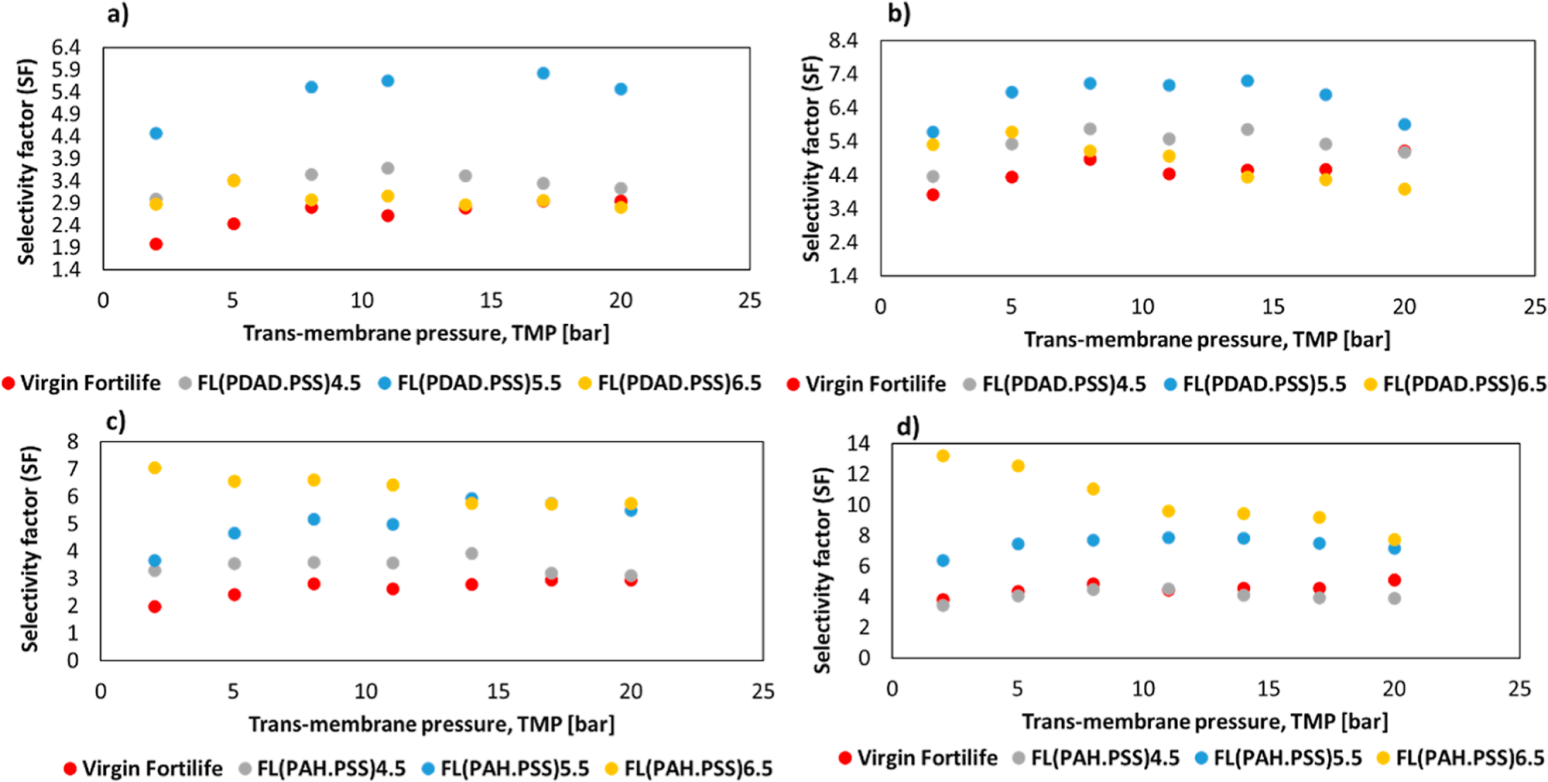
Selectivity factor of the synthetic solution at increasing TMP: (a) Na/Ca and (b) Na/Mg for PDADMAC/PSS-modified membranes and (c) Na/Ca and (d) Na/Mg for PAH/PSS-modified membranes.

## 4 Conclusions

This study demonstrated the effectiveness of layer-by-layer (LBL) surface modification in active surface characteristics and morphology. Using a semiaromatic polyamide NF membrane (Fortilife-XN) as a substrate, modifications with (PDADMAC/PSS) and (PAH/PSS) multilayers resulted in significant changes in key membrane properties, such as wettability, surface roughness, and thickness control. Detailed characterization using advanced techniques—including FTIR, AFM, FE-SEM, ellipsometry, and Raman imaging—provided valuable insights into the structural and chemical alterations induced by LBL modifications. Membranes modified with PDADMAC showed smoother and more hydrophilic surfaces, whereas PAH layers formed bulkier structures, leading to an increased thickness. Ellipsometry was crucial for precise measurement of the nanometer-scale thickness of the modified layers, and Raman imaging proved that it can be an appropriate method to observe the multilayer modification on a nanometer scale in the cross section while providing interesting information about the uniform deposition of polyelectrolytes, which is essential for consistent membrane performance. These findings underscore the importance of optimizing positively charged terminal layers to minimize fouling and extend the membrane lifespan. This research lays the foundation for designing next-generation NF membranes that achieve high permeability while maintaining excellent selectivity, supporting applications in water treatment, scaling control, and selective ion separation. (PDADMAC/PSS)­4.5 and (PAH/PSS)­1.5 emerged as particularly promising, demonstrating an ideal balance of wettability and surface roughness to enhance operational efficiency and reduce fouling with permeability around 10 L/(m^2^ h bar).

The preliminary testing results revealed that the bare membrane showed a sulfate rejection of 97%, a Mg^2+^ rejection of 88%, a Ca^2+^ rejection of 79%, and a Na^+^ rejection of 48%. After modification, (PDADMAC/PSS) 5.5 achieved a 99% sulfate rejection, 93% Mg^2+^, and 90% Ca^2+^, with Na^+^ rejection slightly increasing. PAH 6.5 reached over a 99% sulfate rejection, 95% Mg^2+^, and 92% Ca^2+^, with a Na^+^ rejection of 51%. The average Na/Ca selectivity for the bare membrane was 2.6 ± 0.4, improving to 5.4 ± 0.5 for (PDADMAC/PSS)­5.5 and to 6.1 ± 1.5 for PAH 6.5, whereas the average Na/Mg selectivity increased from 4.5 ± 0.3 to 6.7 ± 0.6 for (PDADMAC/PSS) 5.5 and to 10.4 ± 1.9 for PAH 6.5. All in all, this work contributes innovative methodologies and valuable insights for advancing in membrane technology and fostering sustainable water management practices.

## Supplementary Material



## Notes

Details on the effect of tuning parameters on LBL coating, morphological images of the active surface as the number of BLs increases, and Raman spectra of virgin and 6.5 BL-modified membranes (PDF)
