# Supplementary material for: Optimizing Nanofiltration Membrane Layer-by-Layer Modification: Chemometric and Morphological Insights
Source: ACS Appl Polym Mater. 2025 Jun 26;7(13):8471–85. doi: 10.1021/acsapm.5c00815 (PMC12261275; doi:10.1021/acsapm.5c00815)
Supplement: Supplementary file 1 [file ap5c00815_si_001.pdf]

Supporting Information

Optimizing nanofiltration membrane layer-by-layer modification: chemometric and morphological insights

Tanaz Moghadamfar[a,b], Rodrigo Rocha de Oliveira[c], José Luis Cortina[a,b,c], Luis J.del Valle[a,b], Anna de Juan[c], Mònica Reig[a,b,*]

[a]Chemical Engineering Department, Escola d’Enginyeria de Barcelona Est (EEBE), Universitat Politècnica de Catalunya (UPC)-Barcelona TECH, Campus Diagonal-Besòs, 08930 Barcelona, Spain

[b]Barcelona Research Center for Multiscale Science and Engineering, Campus Diagonal-Besòs, 08930 Barcelona, Spain

[c]Chemometrics group, Universitat de Barcelona, Dept. of Chemical Engineering and Analytical Chemistry, Martí I Franquès 1, 08028 Barcelona, Spain

[d]CETaqua, Carretera d’Esplugues, 75, 08940 Cornellà de Llobregat, Spain

* Corresponding author: monica.reig@upc.edu

Supporting Information

A) The properties and performance of the resulting multilayered films are primarily determined by the *concentration of polyelectrolytes* used in LBL modification, and careful optimization of polyelectrolyte concentration is frequently required to achieve desirable film characteristics. This is so that thicker layers may be deposited since a higher concentration allows more material to be adsorbed onto the substrate in each layer [1]. Higher poly electrolyte (PE) concentrations have been linked to an increase in layer thickness, according to several investigations; however, PE adsorption may have particular limitations [1–3]. Further, PE chains interacting with the surface beneath can lead to fewer binding sites per PE chain, which is the source of the rise in PE adsorption. As a result, with greater PE concentrations, fewer intrinsic connections emerge [4].

B) The PE coating solutions' *salt content* controls the PE chain conformation and layer adsorption (**Figure S1**). The development and characteristics of the multilayered films can be strongly influenced by the salt concentration in the solution, often known as the ionic strength [5–7]. Low salt concentrations tend to cause counterions to stay free in solution, which causes intrachain repulsion to cause an elongated PE chain[8]. The counterions at high salt concentrations screen the PE charges, reducing the repulsive contact between the charged PE groups. A more flexible, coiled shape is produced due to decreased repulsion [8]. Moreover, the salt concentration is raised by releasing counterions, which lowers the entropy. In other words, there are more salt ions in the solution as the concentration of salt rises. These interactions between the salt ions effectively neutralize the charged groups on the PE chains. Consequently, the solution has fewer counterions, which lowers the system's entropy gain [9]. Layers will form without interaction between the PEs; otherwise, the PEs remain in the solution. The solubility of PE or the breakdown of the PE layer at high salt concentrations limits the maximum salt concentration employed [10]. Lastly, a greater concentration of salt causes the PEs to swell more. Higher salt concentrations typically lead to more significant amounts of free, extrinsically compensated charges. These charges interact more with water, causing higher degrees of swelling [6,10]. As a result, adding more salt to the coating solution within the range of acceptable salt concentrations increases the thickness of PE layers. Polyelectrolyte aggregation, insufficient coverage, and uneven film development can be caused by either an excessively low or high salt concentration [11,12].

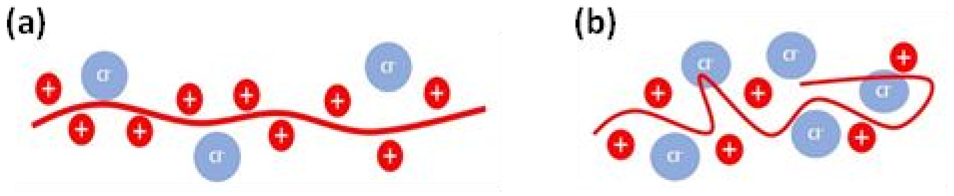

Figure S1. Effect of salt concentration on the structure of the polyelectrolyte chain a) low salt concentration b) high salt concentration.

C) The *kind of salt* and how well-hydrated it influences the creation of layers and salt concentration during PE coating. Generally speaking, PE layers adsorbed with less hydrated ions (large ions) are thicker than those adsorbed with more hydrated ions (small ions) [13–15]. Highly hydrated anions reduce the freedom of motion of PEs because they attach water molecules firmly, strengthening the hydrogen bond [15]. The converse is valid for the least hydrated anions, whose weak electrostatic fields, high polarizability, and low electronegativity cause the hydrogen bonding between the solvent molecules and ions to become unstable. Because they bind to the poly cations (PCs) and cause PC coiling, using low-hydrated anions lowers the charge density of the PCs. Using additional anions raises the amount of extrinsic charge compensation between the PE layers, much as increasing salt concentration does. The creation of thicker layers due to the PE coiling and extrinsic charge correction reduces the membrane's water permeability [15].

D) The assembly is affected by the *kind of polyelectrolyte* employed in LBL because of the charge's accessibility, density, and hydrophobicity in the PE [16]. Typical PCs comprise an ammonium group with various substituents, ranging from primary to quaternary ammonium groups. The PCs poly(diallyl dimethylammonium chloride) (PDADMAC) [10,16,17] and poly(allylamine) hydrochloride (PAH) [18–21], the poly anions (PAs) poly(sodium 4-styrstyrene sulfonate) PSS [10,16,17] and polyacrylic acid (PAA) [22,23] have been studied the most for LBL membranes. The PE couple's hydrophilicity indicates the strength of the intrinsic connections. A strongly coupled PE pair has been established, as evidenced by the low water content of its PC/PA repeating unit [21]. Furthermore, the degree of swelling varies across the PEs according to the hydrophilicity of the PE complex. According to Dubas et al. (2001), the swelling degree of the PDADMAC/PSS pair is reported to be higher than PAH/PSS, and among all, PDADMAC/PAA has the highest degree of swelling [6]. PE pairs with higher hydrophobic complexes bind together more firmly and cause less swelling [6,24].

E) The *charge density* (CD) of the PEs, which is the number of charges per carbon atom in the monomer unit and consequently indicates the amount of charge that a PE is capable of holding, is one of the most significant control parameters. While the charge of weak PEs is heavily dependent on the pH of the solution because it establishes the degree of ionization, the CD of strong PEs is pH-independent. Consequently, the structure and characteristics of the PE layer, including its charge, thickness, growth pattern, and interdiffusion of layers, are determined by a slight change in the coating solution's pH [25,26]. A weak PA has the maximum ionization degree at a low pH, whereas a weak PC has the opposite effect. A stretched and stiff chain conformation results from the maximum ionization degree, which raises the electrostatic repulsive forces between the various PE chain segments [27]. It follows that fewer PE chains can adsorb on the surface because a single chain might compensate for more accessible surface charges. Thin layers form as a result of the chains' stretched conformation. For fully charged polyelectrolytes, linear adsorption is typically seen, as the creation of an inherent bond is both entropically and kinetically preferred between the PE chains [28]. The electrostatic force that repels one segment of the chain from the other reduces with decreasing ionization degree, leading to a coiled chain conformation on the surface and thicker but more open layers. Since there is less electrostatic repulsion between the adsorbing

PE and the underlying PE layers, thicker PE layers are generated in addition to the PE coiling [29].

F) The adsorbed *final layer* significantly influences the membrane performance in addition to the kind of PEs utilized during layer creation. This terminal layer affects the membrane's charge, degree of swelling, water mobility, and contact angle, among other properties [5,29]. Odd-even effects refer to the variations between PC and PA-terminated membranes. When PC-terminated, a membrane is more positively charged; when it is PA-terminated, the membrane is more negatively charged. Because of the interdiffusion of the PE layers, complete charge reversal is not always present but can occasionally happen [16]. Furthermore, the different PEs have different degrees of swelling. For example, PDADMAC has a four-fold greater capacity to swell than PSS [10]. Whether the PE layers form in the pore-dominated or layer-dominated regimes determines the precise impact of PE swelling [5]. The PE layers are primarily covered inside the support's pores while in the pore-dominated regime. In that instance, the larger swelling of PDADMAC, which narrows the pores, causes membranes coated with PSS-terminating layers to result in a higher flux than the PDADMAC-terminating layers. The opposite is true in the layer-dominating regime. Because PDADMAC swells more than PSS, the resulting layer is thicker but more open, and its water permeability is higher as a result [30]. Moreover, the hydrophilicity of the ended layers may vary. When compared to hydrophobic surfaces, surfaces with higher hydrophilicity typically have higher permeabilities. Membranes that are finished with PSS layers, as opposed to PDADMAC, are recognized to be more hydrophilic. However, because PDADMAC has a greater swelling degree, this impact is usually ignored [29,31]. Positively ended PEMMs make it possible to increase their applicability, decrease fouling, and improve capillary effects. The deliberate insertion of positively charged layers attracts water molecules, increasing the capillary force in the membrane matrix. Fluid flow across the membrane thus becomes more streamlined and effective. Furthermore, the membranes positively charged surface acts as an electrostatic repulsion to prevent negatively charged foulants from adhering to it, extending its operating life and maintaining peak performance over time [32,33].

G) Moreover, for the positive charge-ended membranes, the *pH* of PCs is an essential factor that affects membrane thickness [29]. Under primary conditions with low polycation charge, the thickest films are formed. This is attributed to the ability of polycation chains to assume more coiled conformations due to reduced electrostatic repulsion between segments. This coiled conformation persists during film formation, facilitated by weaker electrostatic repulsion between adsorbing PE molecules, further forming thicker films. Conversely, powerfully charged chains adopt stretched, rod-like conformations due to repulsion between charged segments. This leads to flatter PE molecule adsorption, forming thinner multilayers, particularly under lower pH conditions [29]. It is crucial to consider that for modified membranes with thin layers, their ion rejections predominantly rely on size exclusion, whereas for thicker layers, they are based on Donnan exclusion [5].

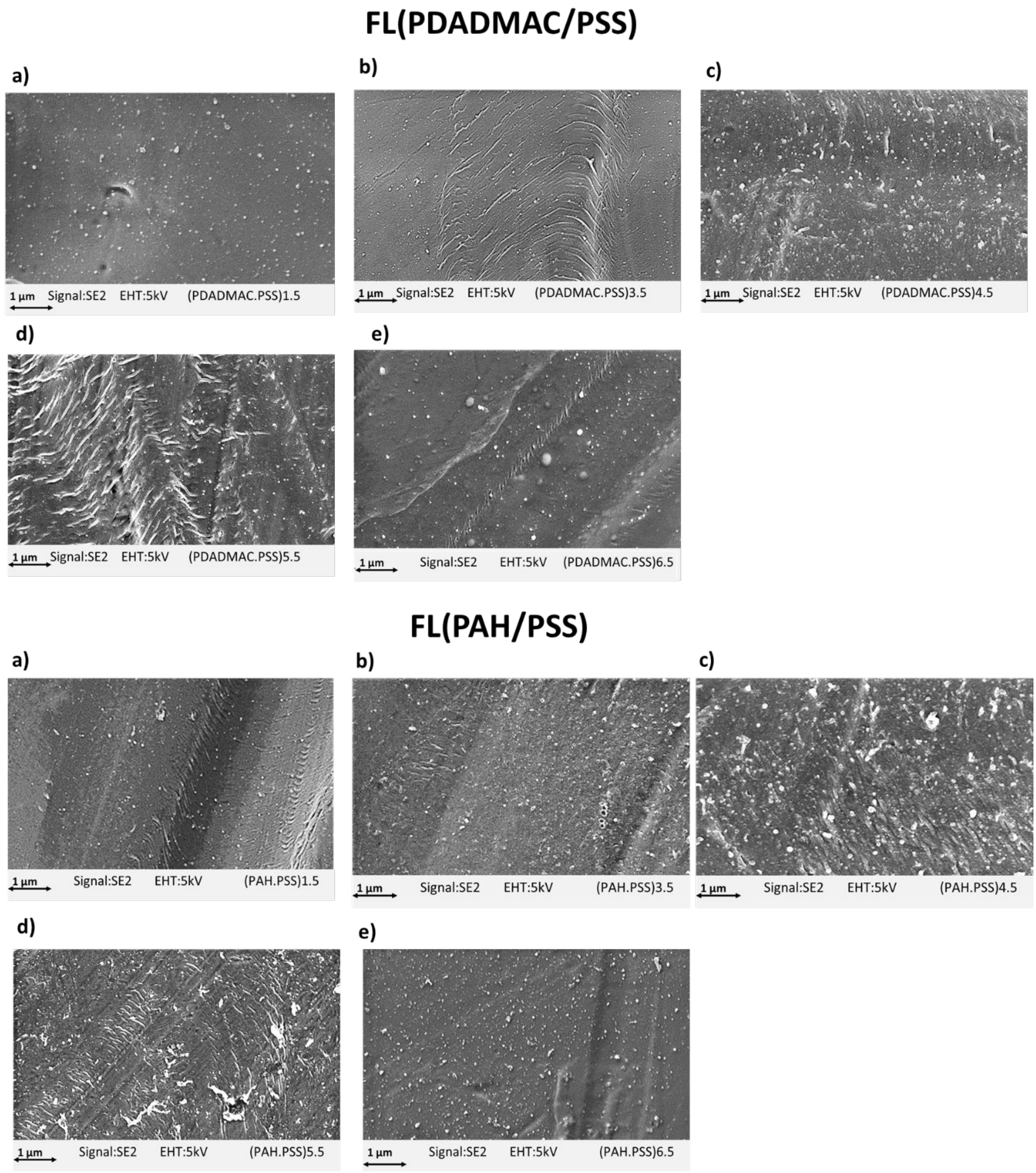

Figure S2. Surface FE-SEM images of modified membranes with (PDADMAC/PSS) (upper part) and (PAH/PSS) (below part) by increasing the number of layers from 1.5 to 6.5 BLs (a-e).

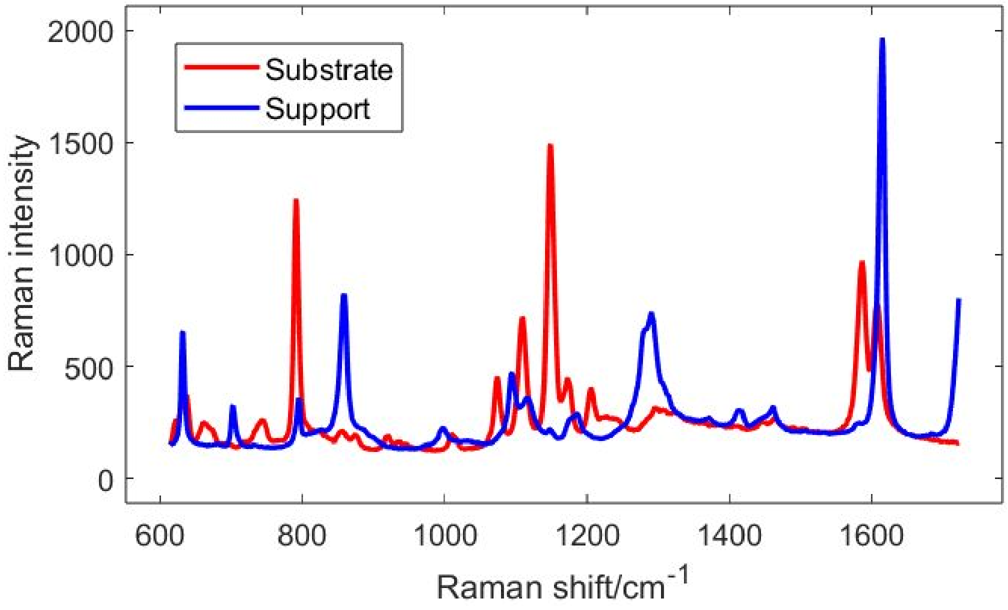

Figure S3. Raman intensity spectra as a function of the Raman shift of the substrate (in red) and support (in blue) layer of raw Fortilife membrane.

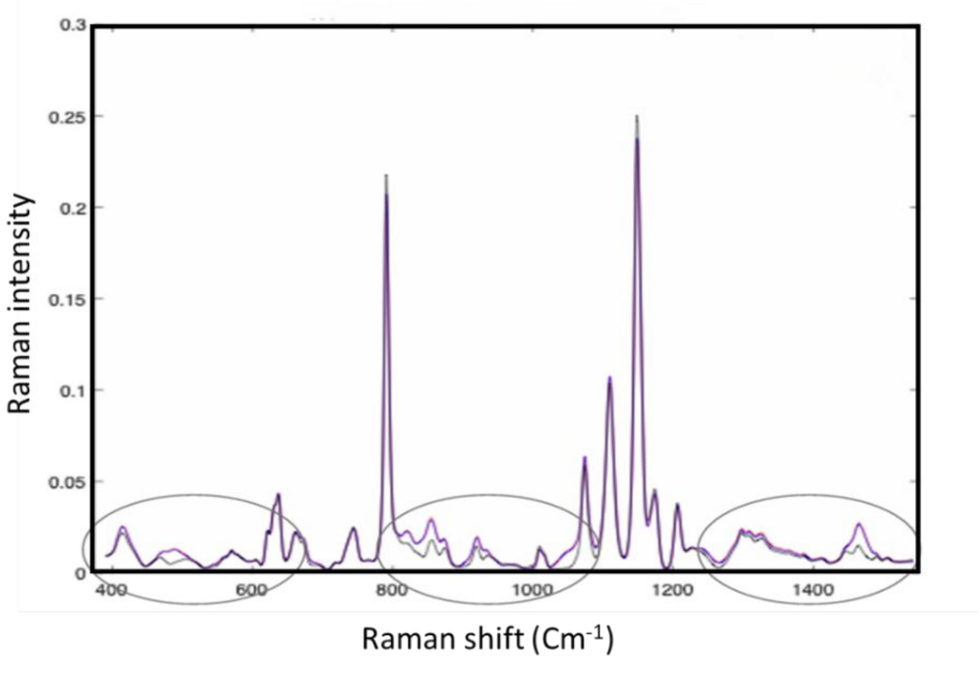

Figure S4. Average Raman spectra of raw membrane (in black) and modified membranes FL(PAH/PSS)6.5 (in red) and FL(PDADMAC/PSS)6.5 (in blue).

(1) Voigt, U.; Jaeger, W.; Findenegg, G. H.; Klitzing, R. V. Charge Effects on the Formation of Multilayers Containing Strong Polyelectrolytes. *Journal of Physical Chemistry B* **2003**, *107* (22), 5273–5280. https://doi.org/10.1021/jp0256488.

(2) Wang, L.; Fu, Y.; Wang, Z.; Fan, Y.; Zhang, X. Investigation into an Alternating Multilayer Film of Poly(4-Vinylpyridine) and Poly(Acrylic Acid) Based on Hydrogen Bonding. *Langmuir* **1999**, *15* (4), 1360–1363. https://doi.org/10.1021/la981181+.

(3) Porus, M.; Maroni, P.; Borkovec, M. Response of Adsorbed Polyelectrolyte Monolayers to Changes in Solution Composition. *Langmuir* **2012**, *28* (50), 17506–17516. https://doi.org/10.1021/la303937g.

(4) Garg, A.; Heflin, J. R.; Gibson, H. W.; Davis, R. M. Study of Film Structure and Adsorption Kinetics of Polyelectrolyte Multilayer Films: Effect of PH and Polymer Concentration. *Langmuir* **2008**, *24* (19), 10887–10894. https://doi.org/10.1021/la8005053.

(5) de Grooth, J.; Oborný, R.; Potreck, J.; Nijmeijer, K.; de Vos, W. M. The Role of Ionic Strength and Odd-Even Effects on the Properties of Polyelectrolyte Multilayer Nanofiltration Membranes. *J Memb Sci* **2015**, *475*, 311–319. https://doi.org/10.1016/j.memsci.2014.10.044.

(6) Dubas, S. T.; Schlenoff, J. B. Swelling and Smoothing of Polyelectrolyte Multilayers by Salt. *Langmuir* **2001**, *17* (11), 7725–7727.

(7) Joseph, N.; Ahmadiannamini, P.; Hoogenboom, R.; Vankelecom, I. F. J. Layer-by-Layer Preparation of Polyelectrolyte Multilayer Membranes for Separation. *Polym Chem* **2014**, *5* (6), 1817–1831. https://doi.org/10.1039/c3py01262j.

(8) Zan, X.; Peng, B.; Hoagland, D. A.; Su, Z. Polyelectrolyte Uptake by PEMs: Impact of Salt Concentration. *Polym Chem* **2011**, *2* (11), 2581–2589. https://doi.org/10.1039/c1py00280e.

(9) Springer Berlin, H. *Polyelectrolyte Complexes in the Dispersed and Solid State II*; Martin Müller, Ed.; Springer Berlin, Heidelberg, 2014. https://doi.org/https://doi.org/10.1007/978-3-642-40746-8.

(10) Miller, M. D.; Bruening, M. L. Correlation of the Swelling and Permeability of Polyelectrolyte Multilayer Films. *Chemistry of Materials* **2005**, *17* (21), 5375–5381. https://doi.org/10.1021/cm0512225.

(11) Guzmán, E.; Ritacco, H.; Rubio, J. E. F.; Rubio, R. G.; Ortega, F. Salt-Induced Changes in the Growth of Polyelectrolyte Layers of Poly(Diallyl-Dimethylammonium Chloride) and Poly(4-Styrene Sulfonate of Sodium). *Soft Matter* **2009**, *5* (10), 2130–2142. https://doi.org/10.1039/b901193e.

(12) Schoeler, B.; Kumaraswamy, G.; Caruso, F. Investigation of the Influence of Polyelectrolyte Charge Density on the Growth of Multilayer Thin Films Prepared by the Layer-by-Layer Technique. *Macromolecules* **2002**, *35* (3), 889–897. https://doi.org/10.1021/ma011349p.

(13) Casimiro, A.; Weijers, C.; Scheepers, D.; Borneman, Z.; Nijmeijer, K. Kosmotropes and Chaotropes: Specific Ion Effects to Tailor Layer-by-Layer Membrane Characteristics and Performances. *J Memb Sci* **2023**, *672* (2023). https://doi.org/10.1016/j.memsci.2023.121446.

(14) Salomäki, M.; Tervasmäki, P.; Areva, S.; Kankare, J. The Hofmeister Anion Effect and the Growth of Polyelectrolyte Multilayers. *Langmuir* **2004**, *20* (9), 3679–3683. https://doi.org/10.1021/la036328y.

(15) Hribar, B.; Southall, N. T.; Vlachy, V.; Dill, K. A. How Ions Affect the Structure of Water. *J Am Chem Soc* **2002**, *124* (41), 12302–12311. https://doi.org/10.1021/ja026014h.

(16) Ouyang, L.; Malaisamy, R.; Bruening, M. L. Multilayer Polyelectrolyte Films as Nanofiltration Membranes for Separating Monovalent and Divalent Cations. *J Memb Sci* **2008**, *310* (1–2), 76–84. https://doi.org/10.1016/j.memsci.2007.10.031.

(17) SU, B.; Wang, T.; Wang, Z.; Gao, X.; Gao, C. Preparation and Performance of Dynamic Layer-by-Layer PDADMAC/PSS Nanofiltration Membrane. *J Memb Sci* **2012**, *423–424*, 324–331. https://doi.org/10.1016/j.memsci.2012.08.041.

(18) Junker, M. A.; Regenspurg, J. A.; Valdes Rivera, C. I.; Brinke, E. te; De Vos, W. M. Effects of Feed Solution PH on Polyelectrolyte Multilayer Nanofiltration Membranes. *ACS Appl Polym Mater* **2022**. https://doi.org/10.1021/acsapm.2c01542.

(19) Kamp, J.; Emonds, S.; Wessling, M. Designing Tubular Composite Membranes of Polyelectrolyte Multilayer on Ceramic Supports with Nanofiltration and Reverse Osmosis Transport Properties. *J Memb Sci* **2021**, *620* (November 2020), 118851. https://doi.org/10.1016/j.memsci.2020.118851.

(20) Luo, J.; Dong, C.; He, R.; Liu, C.; He, T. Impact of Support Pore Properties on the Performance of Layer-by-Layer Self-Assembly Nanofiltration Membrane. *Desalination* **2023**, *557* (March), 116596. https://doi.org/10.1016/j.desal.2023.116596.

(21) Reurink, D. M.; Willott, J. D.; Roesink, H. D. W.; De Vos, W. M. Role of Polycation and Cross-Linking in Polyelectrolyte Multilayer Membranes. *ACS Appl Polym Mater* **2020**, *2* (11), 5278–5289. https://doi.org/10.1021/acsapm.0c00992.

(22) Elshof, M. G.; de Vos, W. M.; de Grooth, J.; Benes, N. E. On the Long-Term PH Stability of Polyelectrolyte Multilayer Nanofiltration Membranes. *J Memb Sci* **2020**, *615* (August), 118532. https://doi.org/10.1016/j.memsci.2020.118532.

(23) Ilyas, S.; Joseph, N.; Szymczyk, A.; Volodin, A.; Nijmeijer, K.; de Vos, W. M.; Vankelecom, I. F. J. Weak Polyelectrolyte Multilayers as Tunable Membranes for Solvent Resistant Nanofiltration. *J Memb Sci* **2016**, *514*, 322–331. https://doi.org/10.1016/j.memsci.2016.04.073.

(24) Fu, J.; Fares, H. M.; Schlenoff, J. B. Ion-Pairing Strength in Polyelectrolyte Complexes. *Macromolecules* **2017**, *50* (3), 1066–1074. https://doi.org/10.1021/acs.macromol.6b02445.

(25) Izumrudov, V.; Sukhishvili, S. A. Ionization-Controlled Stability of Polyelectrolyte Multilayers in Salt Solutions. *Langmuir* **2003**, *19* (13), 5188–5191. https://doi.org/10.1021/la034360m.

(26) Schönhoff, M.; Bieker, P. Linear and Exponential Growth Regimes of Multilayers of Weak Polyelectrolytes in Dependence on PH. *Macromolecules* **2010**, *43* (11), 5052–5059. https://doi.org/10.1021/ma1007489.

(27) Curtis, K. A.; Miller, D.; Millard, P.; Basu, S.; Horkay, F.; Chandran, P. L. Unusual Salt and PH Induced Changes in Polyethylenimine Solutions. *PLoS One* **2016**, *11* (9), 1–20. https://doi.org/10.1371/journal.pone.0158147.

(28) Schlenoff, J. B.; Dubas, S. T. Mechanism of Polyelectrolyte Multilayer Growth: Charge Overcompensation and Distribution. *Macromolecules* **2001**, *34* (3), 592–598. https://doi.org/10.1021/ma0003093.

(29) Elzbieciak, M.; Kolasinska, M.; Warszynski, P. Characteristics of Polyelectrolyte Multilayers: The Effect of Polyion Charge on Thickness and Wetting Properties. *Colloids Surf A Physicochem Eng Asp* **2008**, *321* (1–3), 258–261. https://doi.org/10.1016/j.colsurfa.2008.01.036.

(30) Zerball, M.; Laschewsky, A.; Von Klitzing, R. Swelling of Polyelectrolyte Multilayers: The Relation Between, Surface and Bulk Characteristics. *Journal of Physical Chemistry B* **2015**, *119* (35), 11879–11886. https://doi.org/10.1021/acs.jpcb.5b04350.

(31) Ng, L. Y.; Mohammad, A. W.; Rohani, R.; Hairom, N. H. H. Development of a Nanofiltration Membrane for Humic Acid Removal through the Formation of Polyelectrolyte Multilayers That Contain Nanoparticles. *Desalination Water Treat* **2016**, *57* (17), 7627–7636. https://doi.org/10.1080/19443994.2015.1029009.

(32) Zhang, H. Z.; Xu, Z. L.; Ding, H.; Tang, Y. J. Positively Charged Capillary Nanofiltration Membrane with High Rejection for Mg2 + and Ca2 + and Good Separation for Mg2 + and Li +. *Desalination* **2017**, *420*, 158–166. https://doi.org/10.1016/j.desal.2017.07.011.

(33) Mu, S.; Wang, S.; Liang, S.; Xiao, K.; Fan, H.; Han, B.; Liu, C.; Wang, X.; Huang, X. Effect of the Relative Degree of Foulant "Hydrophobicity" on Membrane Fouling. *J Memb Sci* **2019**, *570–571* (October 2018), 1–8. https://doi.org/10.1016/j.memsci.2018.10.023.
